# APOE4 genotype and aging impair injury-induced microglial behavior in brain slices, including toward Aβ, through P2RY12

**DOI:** 10.1186/s13024-024-00714-y

**Published:** 2024-03-11

**Authors:** Jordy Sepulveda, Jennifer Yejean Kim, Joseph Binder, Stefano Vicini, G. William Rebeck

**Affiliations:** 1 Department of Pharmacology & Physiology, Georgetown University, Washington, DC 20007 USA; 2 Department of Neuroscience, Georgetown University, Washington, DC 20007 USA

**Keywords:** Microglia, APOE4, Aging, Alzheimer’s disease, P2RY12, *Ex-vivo* imaging

## Abstract

Microglia are highly dynamic cells that play a critical role in tissue homeostasis through the surveillance of brain parenchyma and response to cues associated with damage. Aging and *APOE4* genotype are the strongest risk factors for Alzheimer’s disease (AD), but how they affect microglial dynamics remains unclear. Using ex vivo confocal microscopy, we analyzed microglial dynamic behaviors in the entorhinal cortex (EC) and hippocampus CA1 of 6-, 12-, and 21-month-old mice *APOE3* or *APOE4* knock-in mice expressing GFP under the *CX3CR1* promoter. To study microglia surveillance, we imaged microglia baseline motility for 20 min and measured the extension and retraction of processes. We found that *APOE4* microglia exhibited significantly less brain surveillance (27%) compared to *APOE3* microglia in 6-month-old mice; aging exacerbated this deficit. To measure microglia response to damage, we imaged process motility in response to ATP, an injury-associated signal, for 30 min. We found *APOE4* microglia extended their processes significantly slower (0.9 µm/min, *p* < 0.005) than *APOE3* microglia (1.1 μm/min) in 6-month-old animals. *APOE*-associated alterations in microglia motility were observed in 12- and 21-month-old animals, and this effect was exacerbated with aging in *APOE4* microglia. We measured protein and mRNA levels of P2RY12, a core microglial receptor required for process movement in response to damage. We found that *APOE4* microglia express significantly less P2RY12 receptors compared to *APOE3* microglia despite no changes in P2RY12 transcripts. To examine if the effect of *APOE4* on the microglial response to ATP also applied to amyloid β (Aβ), we infused locally Hi-Lyte Fluor 555-labeled Aβ in acute brain slices of 6-month-old mice and imaged microglia movement for 2 h. *APOE4* microglia showed a significantly slower (*p* < 0.0001) process movement toward the Aβ, and less Aβ coverage at early time points after Aβ injection. To test whether P2RY12 is involved in process movement in response to Aβ, we treated acute brain slices with a P2RY12 antagonist before Aβ injection; microglial processes no longer migrated towards Aβ. These results provide mechanistic insights into the impact of *APOE4* genotype and aging in dynamic microglial behaviors prior to gross Aβ pathology and could help explain how APOE4 brains are more susceptible to AD pathogenesis.

**Supplementary Information:**

The online version contains supplementary material available at 10.1186/s13024-024-00714-y.

## Introduction

Alzheimer’s disease (AD) is a neurodegenerative disorder characterized by the accumulation of amyloid β (Aβ) plaques, neurofibrillary tangles formation, and cognitive decline [1]. Aging is the strongest risk factor for AD, and the Apolipoprotein E (*APOE*) ε4 allele is the strongest genetic risk factor [2, 3]. Genetic studies have identified a large number of other genetic risk factors for AD, several of which are specifically expressed in microglia [4]. Microglia have a role in Aβ clearance but can also cause chronic inflammation, facilitating or exacerbating AD pathology [5]. Identifying alterations in microglial physiological functions associated with the maintenance of brain homeostasis prior to disease onset may guide toward therapeutic strategies for AD.

Microglia are the resident macrophages of the central nervous system (CNS) that play a pivotal role during neuronal development and maintenance of brain homeostasis [6]. In a healthy brain, microglia express homeostatic membrane receptors that detect chemotactic cues, allowing them to monitor the extraneuronal environment and respond to pathological stimuli [6]. Several mouse models of neurodegeneration identified a subset of disease-associated microglia (DAM) through single-cell and single-nuclei RNA sequencing [5, 7–9]. DAM are characterized by the downregulation of key homeostatic genes associated with microglial surveillance and response to damage [10]. Among the homeostatic microglial genes downregulated is the purinergic receptor P2RY12, which is a core marker of microglia in the healthy brain. P2RY12 is required for the movement of microglial processes in response to chemotactic cues associated with neuronal damage, such as ATP and laser ablation [11, 12]. These microglial transcriptional signatures are associated with chronic AD pathology, but it is necessary to determine how risk factors for AD, such as *APOE4* and aging, affect microglial function during homeostasis and in response to subtle damages in healthy brains.

Microglia rely heavily on the motility of their processes to monitor CNS parenchyma to clear cellular debris and prune dysfunctional synapses. While fixed brain tissue has been informative in studying how *APOE4* affects microglia morphology and interactions with Aβ during AD pathology [5, 13], acute slices are essential in the study of microglial function as they preserve microglial phenotypes in near-physiological conditions [14–16]. Investigating *APOE* genotype and age-associated alterations at baseline and in response to injury in a physiological environment could reveal pathogenic mechanisms associated with AD risk factors. In the current study, we crossed *APOE* knock-in mice (KI) [17, 18] with mice expressing GFP under the CX3CR1 promoter, which tags microglia. We used *ex-vivo* live imaging to measure spontaneous and responsive microglia motility to chemotactic cues associated with neuronal damages, including localized Aβ peptide. We further tested whether *APOE4* was associated with the downregulation of key homeostatic microglial receptor P2RY12. These findings show that *APOE* genotype and aging alter microglia homeostasis and their response to injuries prior to the gross accumulation of AD pathology.

## Materials and methods

### Mice

CX3CR1^GFP/GFP^ mice (JAX stock No. 005582) on a C57BL/6 J (JAX stock No. 000664) [19] were crossed with *APOE3KI* (JAX stock No. 029018) or *APOE4KI* (JAX stock No. 027894) [17] to obtain *APOE3KI:* *CX3CR1*^GFP/−^ and *APOE4KI:* *CX3CR1*^GFP/−^ (referred to in this study simply as *APOE3* and *APOE4* mice). All animals were housed with littermates and kept on a 12-h light/dark cycle with ad libitum access to chow and water. Male and female *APOE3* and *APOE4* mice were sacrificed at 6, 12, and 21 months of age (*n* = 4–5 mice /genotype/sex/age group). Sample sizes are included throughout the methods and in the figure legends. All studies were carried out following the Guide for the Care and Use of Laboratory Animals as adopted by the U.S. National Institute of Health and approved by Georgetown University Animal Care and Use Committee, approval protocol 2016–1160.

### Acute slice preparation

Mice were anesthetized with unmetered isoflurane (Patterson Veterinary) and intracardially perfused with NMDG solution containing N-methyl-D-glucamine (NMDG) 92 mM, KCl 2.5 mM, NaH_2_PO_4_ dihydrate 1.25 mM, NaHCO_3_ 30 mM, HEPES 20 mM, glucose 25 mM, sucrose 10 mM, ascorbic acid 5 mM, thiourea 2 mM, sodium pyruvate 3 mM, N-acetyl-L-cysteine 5 mM, MgSO_4_ heptahydrate 10 mM, and CaCl_2_ dihydrate 0.5 mM at pH 7.3–7.4 and osmolarity 300–310 mOsm/kg. Brains were dissected into two hemispheres, one for *ex-vivo* experiments and the other for immunohistochemistry. For the *ex-vivo* studies, 300 μm horizontal slices, which allow visualization of entorhinal cortex (EC) and hippocampus (CA1), were cut in ice-cold NMDG using the Vibratome 3000 plus Sectioning System. Sections were incubated for 5 min in NMDG at 32 °C, followed by recovery for 30 min at 32 °C in artificial cerebrospinal fluid (aCSF) containing NaCl 120 mM, KCl 3.5 mM, NaH_2_PO_4_ 1.25 mM, NaHCO_3_ 26 mM, CaCl_2_ dihydrate 1 mM, MgCl_2_ 7 mM, and dextrose 10 mM at pH 7.3–7.4 and osmolarity 300–310 mOsm/kg. Slices were transferred to room temperature (22–24 °C) and equilibrated for > 10 min before use. In the case of PSB treatment, acute brain slices were recovered for 30 min in 10 μM of the P2RY12 antagonist PSB-0739 (Cat# 3983, TOCRIS) in aCSF as described in [20]. To limit artifactual microglial activation from sectioning, we adapted the guidelines of electrophysiological investigation of microglia [21]; therefore, all slices were used within 5 h of euthanasia, and all microglia studied had soma at least 30 μm from the cut surface. All experiments were performed in recording aCSF solution containing NaCl 124 mM, KCl 3.5 mM, NaH_2_PO_4_ dihydrate 1.2 mM, NaHCO_3_ 26 mM, CaCl_2_ dihydrate 2 mM, MgCl_2_ 1 mM, and dextrose 10 mM at pH 7.3–7.4 and osmolarity 300–310 mOsm/kg. Recording aCSF solution was maintained at pH 7.4 by bubbling with carbogen gas (95% O_2_ /5% CO_2_, Roberts Oxygen). All experiments were conducted at room temperature.

### Immunofluorescence

One brain hemisphere was fixed in 4% PFA and 4% sucrose, and dehydrated sequentially in 10%, 20%, and 30% sucrose before freezing in cold 2-methylbutane (Cat# O3551-4, Fisher Scientific). Coronal slices (30 μm thick) were cut in the microtome and stored in cryoprotectant. Sections were washed in Tris-buffered saline (TBS, pH 7.4) and blocked in 10% normal goat serum (NGS) + 1% bovine serum albumin in TBS plus 0.25% Triton X (TBSX, pH 7.4) for 1.5 h. Brain slices were treated overnight at 4 °C with gentle agitation with purified rat anti-P2RY12 clone S160017D (1:100, Cat # 848,002, Biolegend), rabbit anti-APOE (1:200, Cat#: 13366, Cell Signaling Technology), mouse anti-GFAP (1:1000, Cat#: 3670, Cell Signaling Technology), rabbit-anti Tmem119 (1:1000, Cat#: PAS-119617, Invitrogen), and rabbit anti-IBA1 (Cat#:019–19741, WAKO) in TBSX with 1% NGS. After three wash steps with TBSX, brain sections were incubated with Alexa fluor 594 conjugated donkey anti-rat (1:1000, Cat# A212209, Invitrogen), Alexa fluor 594 conjugated goat anti-rabbit (1:1000, Cat# A11072, Invitrogen), Alexa fluor 594 conjugated donkey anti-mouse (1:1000, Cat# A21203, Invitrogen), or Alexa fluor 647 conjugated donkey anti-rabbit (1:1000, Cat# 711–605-152, Jackson ImmunoResearch) for 2 h at room temperature.

Total P2RY12 expression in CA1 was calculated as the fluorescent intensity across the entire image (arbitrary units) using Image J. To measure P2RY12 in microglial processes, we outlined the microglial soma, cropped out that portion of the images, and measured the remaining fluorescent intensity. The percent P2RY12 in processes was calculated as fluorescent intensity from processes over the total fluorescent intensity (processes plus soma). We analyzed 2 images from 2 brain slices for each animal (*n* = 4 animals/genotype). Experimenters were blinded to the group type during image analysis.

### Confocal imaging

Confocal Z- and ZT-stacks were taken with a laser scanning microscope system (Thor Imaging System Division) equipped with 488/561/642 nm laser and Green/Red/Far-red filters and mounted on an upright Elipse FN1 microscope (Nikon Instruments). 284 × 284 x 20 μm (xyz) volumes of hippocampal slices containing the CA1 and cortical slices containing the entorhinal cortex (EC) were imaged through the 60 × water-dipping objective (CFI Fluor 60XW, NA = 1.0, WD = 2 mm, Nikon). Differential interference contrast images (on acute or fixed slices) were used to identify and confirm the region of interest as CA1 or EC.

### Microglial density and morphology

For microglia density and morphology quantification, 2048 × 2048-pixel Z-stacks, 20 planes 0.5 μm apart, were taken from 30 μm fixed slices. We analyzed the maximal intensity projections across the z-axis. For microglia density, counts of GFP-positive cell bodies were calculated using Image J from single 284 × 284 x 20 μm fields containing CA1 or EC per hemisection. Microglial morphology was quantified as described [22]. Briefly, fluorescent photomicrographs were transformed into gray-scale images and binarized by setting thresholds. Binarized microglia were then skeletonized, and the endpoint boxels (number of branches) and total branch length (total length of microglia) per individual microglia were measured. We analyzed 2 images from 2 brain slices for each animal (*n* = 4 animals/genotype/sex/age group). Experimenters were blinded to group type during image analysis.

### Microglial process motility at baseline and in response to ATP

For baseline and responsive microglia motility experiments, 1024 × 1024 pixel ZT-stack images from 11 planes 1.5 μm apart were taken every 20 s. When necessary, time lapses were stabilized using the StackReg plugin [23] in Image J [24]. Baseline motility was imaged for 20 min in both the CA1 and EC of naïve slices, or in these regions in the presence of an aCSF-containing pipette (0 mM ATP, the control condition for responsive motility experiments, tip resistance 3–5 m Ω). Time-lapse images were manually cropped, then thresholded and binarized in the region of interest (ROI) (adapted from [25]). Binary ROI from each timepoint t(x) were color-merged with a t(x + 1) ROI, generating images containing red pixels representing retracting structures, green pixels representing extending structures, and yellow pixels representing static structures. The numbers of each colored pixel were counted using the Color Counter plugin (Wayne Rasband in ImageJ. For each pair of frames (t(x) and t(x + 1), we calculated the motility index (number of green pixels + number of red pixels/number of yellow pixels). The motility index of each paired frame (e.g., t0 + t1; t1 + t2; t2 + t3) was averaged for the whole 20-min time-lapse.

For reactive microglia motility, a patch pipette containing 1, 3, or 10 mM ATP in aCSF was lowered into CA1 or EC 30 μm deep; the ATP was allowed to diffuse into the tissue without pressure in the pipette. The surrounding volume was imaged for 30 min. ATP concentration and imaging time were chosen based on previous studies [19]. We quantified microglial process velocity by using the Manual Tracking plugin (Fabrice Cordelières) in ImageJ. Between 3 and 5 processes per microglia were manually tracked moving towards the ATP-containing pipette. If a process reached the pipet tip prior to 30 min, its velocity was only calculated until that time. To determine that process motility was due to microglia responding to ATP and not to the pipette piercing the tissue, a pipette containing 0 mM ATP (aCSF only) was lowered, and the surrounding volume was imaged. No directional motility was elicited to nearby microglia by this control. For all time-lapse analysis, we analyzed one time-lapse image from 1–2 brain slices for each animal (*n* = 4–6 animals/genotype/sex/age group). Experimenters were blinded to group identity during image analysis.

### Quantitative RT-PCR (qRT-PCR)

Cortices from 6-month-old mice (*APOE3* *n* = 6, *APOE4* *n* = 6) were cryo-pulverized, and total RNA was isolated using Trizol plus RNA Purification Kit (Cat # 12,183,555, Invitrogen). cDNA was synthesized using High-capacity cDNA Reverse Transcription kit (Cat # 436,814, Applied Biosystems). cDNA (1:50 dilution, 4 μl) was amplified by real-time PCR using PowerUp SYBR Green Master Mix (Cat # A25742, Applied Biosystems). Samples were standardized to GAPDH. Synthetic oligonucleotides were used for mouse P2RY12 (forward: 5’-TGAAGACCACCAGGCCATTT-3’ and reverse: 5’ AGGCCCAGATGACAACAGAAA-3’) and mouse GAPDH (forward: 5’-GTGTTTCCTCGTCCCGTAGA-3’ and reverse: 5’-ATTCCGTTCACACCGACCTT-3’). Samples were analyzed in triplicate, and RNA levels were reported as fold differences between *APOE4* brains and *APOE3* brains (defined as 1.0). Results were analyzed using the double delta CT method.

### Aβ-42 preparation and injection

HiLyte Fluor 555-labeled Aβ-42 (Cat# AS-60480–01, Anaspec) peptide was used in the experiments related to microglial processes responses to Aβ. HiLyte Fluor 555-labeled Aβ-42 (0.1 mg) was diluted in 1% NH_4_OH to obtain a 1 mg/ml peptide solution. The Aβ-42 solution was diluted in aCSF to 100 μM aliquots and stored at -20 °C for subsequent experiments. The same concentration of fluorescein amidites (5FAM) labeled scrambled Aβ-42 (Cat# AS-60892, Anaspec) was prepared and used as a negative control. The protocol for Aβ injection in acute brain slices was adapted from [26] and [27]. Briefly, 5 μl of Aβ-42 was filled into a glass patch pipette (tip resistance 2–3 mΩ) connected with transparent tubing to a 3 ml syringe. The loaded glass patch pipette was carefully lowered into the EC and maneuvered into the center of the field, then the plunger of the syringe was moved slowly from the 3 ml to 1 ml position (in about 10 s). To avoid bleed-through, 2048 × 2048-pixel Z-stacks, 20 planes 0.5 μm apart, were acquired in separate channels every 15 min for 2 h. Time-lapse images were manually thresholded, binarized, and color-coded (green pixels = microglia, red pixels = Aβ-42, yellow pixels = microglia + Aβ-42) in Image J. The percent coverage was calculated as the area of yellow pixels over the area of the combination of yellow and red pixels. For all time-lapse analysis, we analyzed one image from 1–2 brain slices for each animal (*n* = 4 animals/genotype). Experimenters were blinded for image analysis.

### Statistical analyses

Data are expressed as mean ± SEM. For microglia morphology, cell density, and immunohistochemistry experiments, individual points represent each animal. For spontaneous and responsive microglia motility experiments, each individual point represents each cell. Datapoints were equally distributed across animals, and we did not detect datapoints for an individual animal skewing the results. Data were statistically analyzed by a two-tailed *t*-test, a one-way ANOVA, or a two-way ANOVA, as indicated. *p* < 0.05 was considered statistically significant. When statistical significance was achieved, Sidak’s multiple comparisons were used. Statistical analyses were done using GraphPad Prism 9.0 Software.

## Results

### Microglia density and morphology in control brains are not affected by *APOE* genotype

**Fig. 1:**
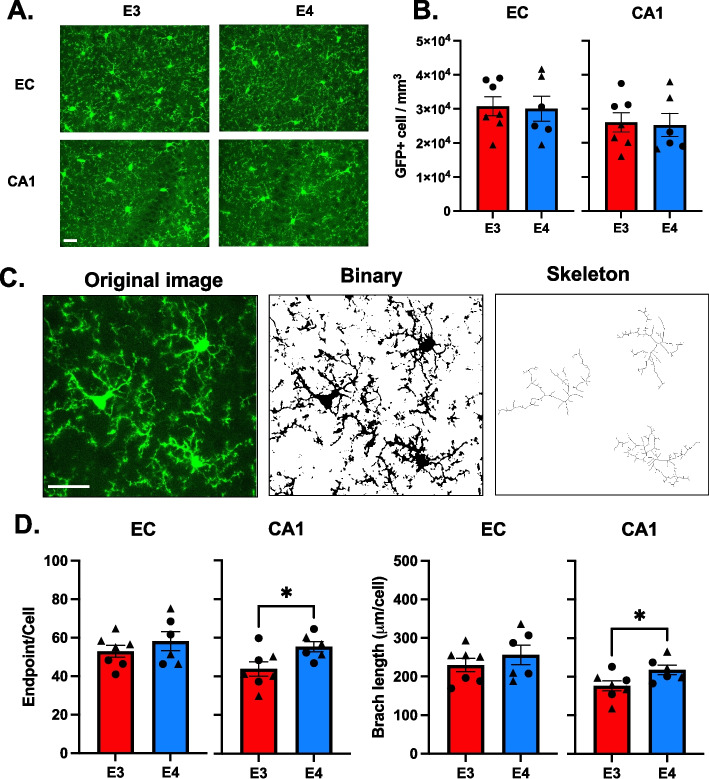
*APOE* genotype does not affect microglia density and morphology. **A** Representative images of microglia in the entorhinal cortex (EC) and hippocampus (CA1) from *APOE3* (E3) and *APOE4* (E4) mice. Scale bar = 50 µm. **B** Quantification of microglia density. Bars represent the mean ± SEM cell density in analyzed fields, and symbols (circles, male; triangle, females) represent data points for each animal. 2–3 brain slices per animal, 4 animals per genotype per sex. **C** Schematic of microglia morphology analysis. Scale bar 25µm. **D** Quantification of microglial endpoint/cell (left) and branch length/cell (right) of the EC and CA1 of E3 and E4 mice. Bars represent the mean ± SEM microglial endpoint/cell and branch length/cell in analyzed fields, and symbols (circles, male; triangle, females) represent data points for each animal. 2–3 brain slices per animal, 4 animals per genotype per sex. *p < 0.05; unpaired two-tailed Student’s t test

Microglia play a central role in CNS homeostasis and respond to different insults by changing morphology, phagocytic activity, and release of cytotoxic and neuroprotective factors [28, 29], with functions that are brain region-dependent [30]. The entorhinal cortex (EC) and hippocampus (CA1) are the brain structures damaged first during AD pathology [31]. Therefore, to define *APOE* genotype-mediated alteration of basic microglial properties, we crossed mice expressing GFP under the CX3CR1 promoter with targeted replacement human *APOE* mice resulting in *CX3CR1*^*GFP/−*^* APOE3* and *CX3CR1*^*GFP/−*^* APOE4*. Microglia-specific markers IBA1 and Tmem119 colocalized with GFP (Figure S1A-B), verifying that the expression of GFP is limited to microglia. Immunostaining colocalized the human APOE protein to astrocytes but not to microglia (Figure S1C), consistent with the reported expression of APOE in control brains [5]. We examined cell density in the EC and CA1 of 6-month-old *CX3CR1*^*GFP/−*^* APOE3* and *CX3CR1*^*GFP/−*^* APOE4* mice (Fig. 1A). Microglia density did not vary between the EC and CA1 regions (Figure S2A) or between *APOE* genotypes (Fig. 1B). Homeostatic microglia are characterized by highly ramified processes that allow the surveillance of brain tissue [32]. To test whether *APOE* genotype modified basic microglial morphology, we examined high-magnification images from 6-month-old *APOE3* and *APOE4* mice (Figure S2B) as described [22] (Fig. 1C). Between *APOE* genotypes, we observed no significant differences in the number of microglial processes (endpoints/per cell) and total branch length in the EC. In the CA1, *APOE4* microglia showed 26% more endpoint/cell (*p* = 0.033, t = 2.242, df = 11, *APOE3*: 43.8 ± 3.7 *n* = 7; *APOE4*: 55.3 ± 2.6, *n* = 6) and 23% more total branches per cell (*p* = 0.042, t = 2.3, df = 11, *APOE3*: 176 ± 13, *n* = 7; *APOE4*: 217 ± 12.2, *n* = 6) relative to *APOE3* (Fig. 1D). Comparing brain regions, we observed that in *APOE3* brains, CA1 microglia exhibited fewer processes (*p* = 0.084, t = 1.88, df = 12) and shorter total branch length (*p* = 0.027, t = 2.50, df = 12) compared to EC microglia (Figure S2C-D). However, this brain-region difference in microglia morphology was not observed in *APOE4* brains.

### *APOE4* genotype reduces microglia surveillance in the entorhinal cortex

**Fig. 2:**
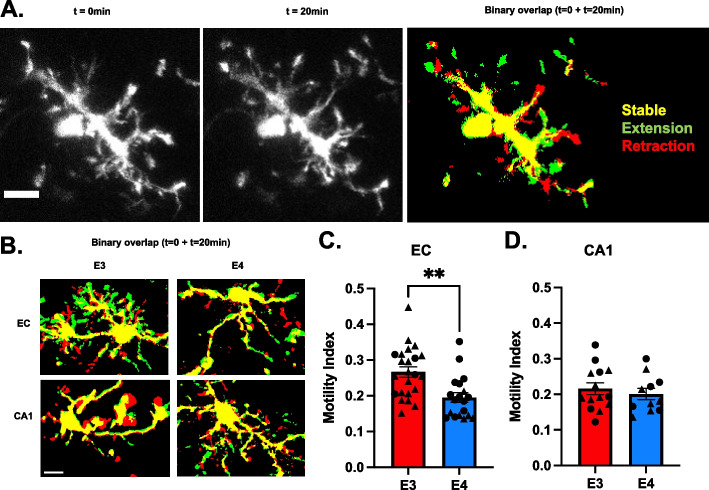
*APOE4* Microglia exhibit lower spontaneous motility in the entorhinal cortex. **A** Confocal images of microglia spontaneous motility time-lapse at different time points, and binary overlap. Scale bar 10 µm. **B** Binary overlaps of APOE3 and APOE4 microglia in the EC and CA1. Quantification of microglial baseline processes movement (motility index = green pixels + red pixels/yellow pixels) in the EC (**C**) and CA1 (**D**). Bars represent the average motility index of all analyzed microglia ± SEM, and symbols (circles, male; triangle, females) represent data points for each microglia (3–4 animals per genotype). ***p < 0.001; unpaired two-tailed Student’s t test

Microglia continuously survey the cerebral microenvironment by extending and retracting their processes throughout the extracellular parenchyma [32, 33]. To investigate how microglial surveillance is affected by *APOE* genotype, we performed *ex-vivo* live imaging of baseline microglia motility for 20 min in acute brain slices from 6-month-old *APOE3* and *APOE4* mice using confocal microscopy (Fig. 2A). Microglia surveillance was quantified as a motility index. Microglia surveillance in EC was significantly lower (30%) in *APOE4* mice (mean motility index = 0.19, *p* = 0.001, t = 3.49, df = 40, *N* = 4 animals, *n* = 19 cells) relative to *APOE3* (mean motility index = 0.27, *N* = 5 animals, *n* = 23 cells) (Fig. 2B-C). This phenotype was not observed in the CA1 area (Fig. 2D). In addition, EC microglia had a 19% higher motility index than CA1 microglia (*p* = 0.032, t = 2.22, df = 35) in *APOE3* brains; *APOE4* microglia did not exhibit brain region-dependent differences in spontaneous motility (Figure S2E). These results suggest that *APOE4* genotype promotes an alteration in spontaneous motility, resulting in the reduction of microglial surveillance.

### *APOE4* genotype reduces microglia responsiveness to ATP

**Fig. 3:**
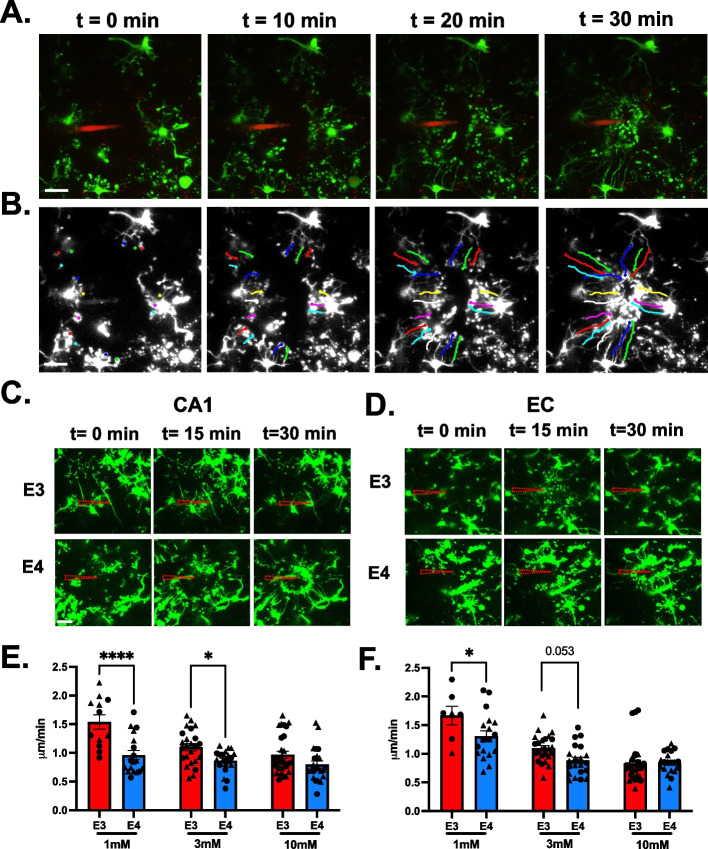
*APOE4* microglia extend their processes in response to ATP slower than *APOE3* microglia. **A** Representative confocal time-lapse showing microglial response (green) to 3 mM ATP in a patch pipette (red) in acute entorhinal slices. Scale bar 20 µm. **B** Illustration of the microglial processes manual tracking over the course of 30 min. Scale bar 20 µm. **C**-**D** *APOE3* (E3) and *APOE4* (E4) microglia responding to patch pipette containing ATP (shown as dashed triangle) in the CA1 (**C**) and EC (**D**). **E**–**F** Processes velocity in the CA1 (**C**) and EC (**D**) in response to 1 mM, 3 mM, and 10 mM ATP. Bar graphs represent the mean velocity ± SEM. Individual points (circles, male; triangle, females) represent the average process velocity (3 processes per cell) of each microglia (3–5 cells per animal). 3–4 animals per genotype per sex (6 months old). Two-way ANOVA with Sidak’s multiple comparison post-hoc analyses *p < 0.05, ****p < 0.0001

ATP is a potent chemotactic cue of neuronal damages, and microglial processes migrate up ATP gradients [32]. We investigated the effect of *APOE* genotype on responsive microglia motility by imaging the extension of processes in response to a patch pipette containing ATP for 30 min (Fig. 3A) and then calculating the velocity through manual tracking (Fig. 3B). Regardless of *APOE* genotype, microglial processes were observed responding to the ATP-filled pipet within one minute in both EC and CA1 processes. *APOE3* microglia converged at the tip of the pipet by 30 min, while *APOE4* microglial processes generally did not reach convergence (Fig. 3 C-D).

In the CA1 region of the hippocampus, the mean process velocity in *APOE4* microglia over the 30 min (0.96 ± 0.08 μm/min, *n* = 18 cells, *N* = 5 animals) was significantly lower (38%) than in *APOE3* microglia (1.54 ± 0.12 μm/min, *n* = 12 cells, *N* = 4 animals, *p* < 0.0001, t = 4.9, df = 121) in response to 1 mM ATP (Fig. 3D). The process movement in response to 3 mM ATP (1.22 ± 0.10 μm/min, *n* = 26 cells, *N* = 6 animals for *APOE3* vs 0.86 ± 0.04 μm/min, *n* = 21 cells, *N* = 6 animals for *APOE4*) revealed a similar 30% lower velocity in *APOE4* microglia (*p* = 0.04, t = 2.5, df = 121) (Fig. 3D). A significant difference in the process velocity between groups was not detected when the pipette contained a higher concentration of ATP (10 mM ATP, 0.97 ± 0.06 μm/min, *n* = 29 cells, *N* = 6 animals for *APOE3* vs 0.8 ± 0.06 μm/min, *n* = 23 cells, *N* = 5 animals for *APOE4*), although a non-significant 18% reduction in *APOE4* mouse brains was observed (*p* = 0.16).

We observed similar results in the EC. There was a statistically significant 22% lower velocity of the *APOE4* microglial processes in response to 1 mM ATP (*p* = 0.03, t = 2.66, df = 116, 1.66 ± 0.16 μm/min, *n* = 7 cells, *N* = 3 animals for *APOE3* vs 1.3 ± 0.09 μm/min, *n* = 18 cells, *N* = 5 animals for *APOE4*) and a 19% lower velocity in response to 3 mM ATP (*p* = 0.053, t = 2.40, df = 116, 1.1 ± 0.05 μm/min, *n* = 25 cells, *N* = 6 animals for *APOE3* vs 0.89 ± 0.05 μm/min, *n* = 21 cells, *N* = 6 animals for *APOE4*). There was not a statistically significant difference in the response to the highest concentration of ATP (10 mM, *p* = 0.99, 0.83 ± 0.06 μm/min, *n* = 31 cells, *N* = 5 animals for *APOE3* vs 0.84 ± 0.04 μm/min, *n* = 20 cells, *N* = 5 animals for *APOE4*) (Fig. 3F).

We observed that process velocity in both *APOE3* and *APOE4* microglia decreased in response to higher ATP concentration, perhaps due to a smaller focal point for microglia to extend the processes up the ATP gradient. Between the brain regions, there were no statistically significant differences in process velocity in response to ATP (e.g., at 3 mM ATP, Figure S2F).

### Aging exacerbates *APOE4*-associated alteration in microglia density and motility

**Fig. 4:**
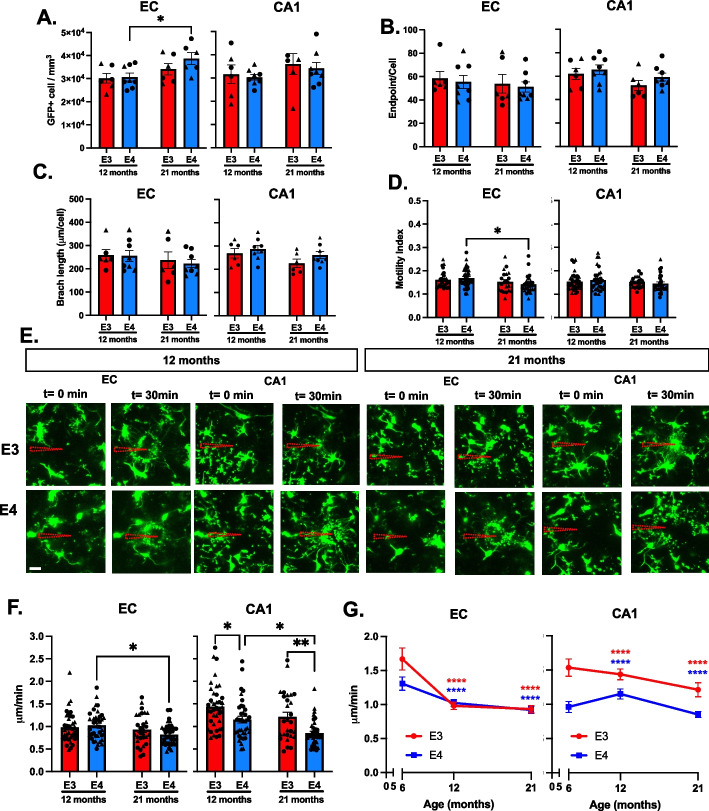
*APOE4*-associated alterations in microglia behaviors are worsened with aging. **A** Quantification of microglia density in EC and CA1 of 12- and 21-month-old mice. Bars represent the mean ± SEM cell density in analyzed fields, symbols (circles, male; triangle females) represent data points for each animal. 2–3 brain slices per animal, 3–4 animals per genotype per sex per age. Two-way ANOVA with Tukey’s multiple comparison post-hoc analyses, *p = 0.014. **B**-**C** Quantification of microglial endpoint/cell (**B**) and branch length/cell (**C**) in the EC and CA1 of 12- and 21-month-old mice. Bars represent the mean ± SEM microglial endpoint/cell and branch length/cell in analyzed fields, and symbols (circles, male; triangle, females) represent data points for each animal. 2–3 brain slices per animal, 3–4 animals per genotype per sex per age. **D** Bars represent the average motility index of all analyzed microglia ± SEM, and points represent data from each microglia 3–4 animals per genotype per sex per age. Two-way ANOVA with Tukey’s multiple comparison post-hoc analyses *p = 0.022. **E** 12- and 21- months old *APOE3* (E3) and *APOE4* (E4) microglia responding to patch pipette containing ATP (shown as dashed triangle) in the CA1 and EC. Scale bar 20 µm. **F** Processes velocity in the CA1 (**C**) and EC (**D**) in response to 1 mM ATP. Bar graphs represent the mean velocity ± SEM. Individual points represent the average process velocity (3 processes per cell) of each microglia (3–5 cells per animal), 3–4 animals per genotype per sex (6 months old). Two-way ANOVA with Tukey’s multiple comparison post-hoc analyses *p < 0.05, **p = 0.006. **G** Comparison of velocity of microglial processes in response to 1 mM ATP in 6-month (shown in Fig. 3), 12-, and 21-month-old mice. Two-way ANOVA with Tukey’s multiple comparison post-hoc analyses; data are compared to 6-month-old mice for either EC or CA1. ****p = 0.0001

To understand how *APOE* genotype impacts homeostatic microglial function during aging, we used 12- and 21-month-old *APOE3* and *APOE4* mice. First, we counted the number of GFP^+^ cells in the EC and CA1 (Figure S3A). We found that *APOE* genotype did not alter microglial density in 12- and 21-month-old brains. However, with aging, microglial density increased by 21% in the EC of *APOE4* mice (*p* = 0.014, 12-month-old: 30,700 ± 1700 GFP^+^ cells vs 21-month-old: 38,700 ± 2600 GFP^+^ cells) but not *APOE3* mice (*p* = 0.233, 12-month-old: 30,100 ± 2100 GFP^+^ cells vs 21-month-old: 34,000 ± 2500 GFP^+^ cells) (Fig. 4A). Second, we quantified microglia morphology (Figure S3B). Our measurements of microglia morphology (endpoints per cell and total number of branches per cell) were not altered by *APOE* genotype or by aging (Fig. 4B and C).

We analyzed the effects of aging on spontaneous microglial motility using 12-month-old and 21-month-old mice. We found that spontaneous motility was lower at these ages than in the 6-month old mice above (Fig. 2). In the EC or the CA1 of older mice, spontaneous microglia motility was not altered by *APOE* genotype (Fig. 4D, and Figure S3C). However, in the EC, the aged, 21-month-old *APOE4* microglia extended and retracted their processes significantly less (16%) relative to 12-month-old *APOE4* microglia (*p* = 0.022, 12-month-old: 0.169 ± 0.006 vs 21-months-old: 0.142 ± 0.006) (Fig. 4D).

We next studied microglial processes movement in response to 1 mM ATP (Fig. 4E). In the CA1 of 12-month-old *APOE4* mice, microglia processes moved 20% slower compared to *APOE3* microglia (*p* = 0.02, *APOE3*: 1.44 ± 0.07 μm/min vs *APOE4*: 1.15 ± 0.07 μm/min). In 21-month-old mice, processes of *APOE4* microglia moved 30% slower compared to *APOE3* microglia (*p* = 0.005, *APOE3*: 1.22 ± 0.54 μm/min vs *APOE4*: 0.85 ± 0.28 μm/min). Moreover, aging exacerbated the reduced motility in response to ATP in *APOE4* microglia (*p* = 0.017), but not in *APOE3* microglia (*p* = 0.17) (Fig. 4F). In the EC, *APOE* genotype did not affect microglia response to ATP. However, we observed that in 12-month-old (*p* < 0.0001, t = 4.7, df = 74, EC 0.98 ± 0.05 μm/min vs CA1 1.44 ± 0.08 μm/min) and 21-month-old (*p* < 0.02, t = 2.36, df = 55, EC 0.93 ± 0.06 μm/min vs CA1 1.2 ± 0.10 μm/min) *APOE3* brains, EC microglia responded to ATP slower than CA1 microglia (Figure S3D-E).

Within *APOE* genotypes, aging affected responsive microglial process motility. In 21-month-old *APOE4* brains, microglia responded to ATP slower than microglia in 12-month-old *APOE4* brains (*p* = 0.02) (Fig. 4F). Graphing these data together, we found in the EC that, between 6-month-old and 21-month-old mice, there was a 47% decrease in processes velocity in *APOE3* brains (*p* < 0.0001, 6-months 1.7 ± 0.1 μm/min vs 21-months 0.93 ± 0.05 μm/min) and 31% decrease in *APOE4* brains (*p* < 0.0001, 6-months 1.3 ± 0.09 μm/min vs 21-months 0.91 ± 0.05 μm/min). In the CA1, there was a 20% decrease in processes velocity in *APOE3* brains (*p* < 0.0001, 6-months 1.5 ± 0.12 μm/min vs 21-months 1.2 ± 0.1 μm/min) and 11% decrease in *APOE4* brains (*p* < 0.0001, 6-months 0.96 ± 0.08 μm/min vs 21-months 0.8 ± 0.04 μm/min) (Fig. 4G). Aging may have a stronger effect on *APOE3* microglia because *APOE4* microglia show an aged phenotype at earlier ages.

### *APOE4* microglia have less P2RY12, a key homeostatic gene associated with motility

**Fig. 5:**
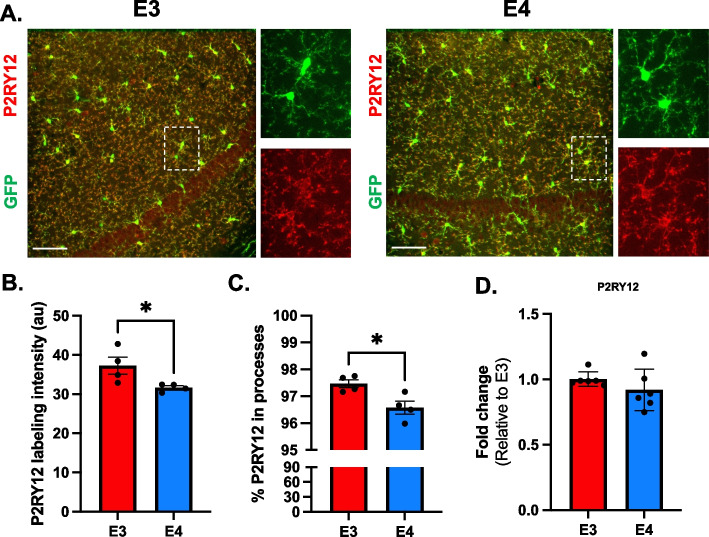
P2RY12 is downregulated in *APOE4* microglia. **A** Confocal images of GFP (green) P2RY12 (red) in *APOE3* (E3) and *APOE4* (E4) brains. P2RY12 is expressed mainly in the membrane of microglial processes distal from microglial soma. Scale bar 60 µm. **B** Quantification of P2RY12 labeling intensity (arbitrate units, see methods) in total field. Individual points represent each animal. 2 brain slices per animal, 4 animals (males) per genotype. *p < 0.05, two-tailed student t-test. **C** Quantification of percent P2RY12 in microglial processes. Individual points represent individual fields. 2 fields per brain slice, 2 brain slices per animal, 4 animals (males) per genotype. *p < 0.05, two-tailed student t-test. Fold change levels of P2RY12 mRNA. RNA was extracted from hemibrain (6 animals per genotype). Level of transcript was determined through double delta CT

To investigate a potential mechanism of the *APOE4-*associated alteration in responsive microglia motility, we examined the homeostatic microglial ATP receptor P2RY12 [12]. We immunostained coronal brain slices from 6-month-old *APOE3* and *APOE4* mice for P2RY12. P2RY12 labeling in the CA1 colocalized only with the cell membrane of microglia, mainly in the processes compared to the cell soma (Fig. 5A) (this co-localization further verifies the expression of GFP as limited to microglia). We found that P2RY12 fluorescent intensity overall in *APOE3* microglia was 16% higher than in *APOE4* microglia (*p* = 0.046, t = 2.51, df = 6, *APOE3* = 37.3 ± 2.2 a.u., *n* = 4 vs *APOE4* = 31.6 ± 0.5 a.u., *n* = 4) (Fig. 5B). Comparing P2RY12 labeling intensity between the microglial soma and processes, we found that nearly all P2RY12 fluorescent intensity was in the processes, but significantly less was in *APOE4* processes compared to the *APOE3* processes (*p* = 0.021, t = 3.1, DF = 6) (Fig. 5C). Through qPCR, we did not detect a difference between *APOE* genotypes in P2RY12 mRNA levels from cerebral cortices of six-month-old mice (Fig. 5D). Thus, we hypothesize that the *APOE4* genotype affected the stability and subcellular distribution of P2RY12 rather than the gene transcription.

### The response to Aβ-42 requires P2Y12 and is reduced in *APOE4* microglia

**Fig. 6:**
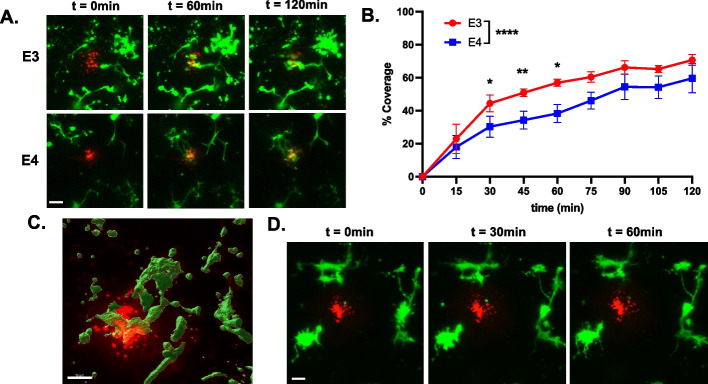
*APOE4* microglia exhibit slower response to Aβ peptides, which requires P2RY12. **A** Representative confocal zt-stacks showing the time course of microglial response (green) to Hi-Lyte Fluor 555-labeled Aβ (Aβ − 42) (red) in acute entorhinal slices. Scale bar 10 µm. **B** Percent coverage computed as the percent area of infused Aβ − 42 covered by microglial processes over time. Mean percent coverage ± SEM plotted. Two-way ANOVA with Sidak’s multiple comparison post-hoc analyses to show the effect of *APOE* genotype, and two-tailed student t-test to compare at each time point. **C** 3D reconstruction of microglial processes internalizing Aβ 6 h post-injection. Scale bar 10 µm. **D** Acute brain slices were incubated in 10 µM PSB. Representative confocal zt-stacks showing the time course of microglial response (green) to Hi-Lyte Fluor 555-labeled Aβ (Aβ − 42) (red). Scale bar 10 µm

We examined whether the slower motility of microglial responses toward ATP was recapitulated toward damage more relevant to AD, i.e., Aβ accumulations. We employed *ex-vivo* microglia live imaging as above, but introduced fluorescent synthetic Aβ-42 instead of ATP. The injected Aβ-42 was immediately visible at the injection site (Fig. 6A, time 0 min panels). We took Z-stack images every 15 min over the course of two hours (Fig. 6A). Processes from surrounding microglia responded to the Aβ-42 within 15 min, and, in general, processes from several microglia reached the Aβ-42 by two hours. The percent coverage was computed as the percent area of Aβ-42 covered by the microglial process over time. *APOE3* microglia were able to reach Aβ-42 within the first 45 min and formed a lamellipodia structure that surrounded Aβ-42 for the remainder of the experiment (45–120 min). In contrast. *APOE4* microglia demonstrated decreased capacity to cover Aβ-42. In the first 45 min, *APOE4* microglia covered 34% Aβ-42, while *APOE3* microglia covered 51% of the infusion (*p* = 0.025). Similar results were observed after 60 min (*p* = 0.009, *APOE3*: 48 ± 7.7% coverage vs *APOE4*: 37 ± 6.4% coverage) and 75 min (*p* = 0.049, *APOE3*: 60 ± 3.2% coverage vs *APOE4*: 46 ± 5% coverage) post-infusion. Two hours after infusion, there was no significant difference in Aβ-42 coverage between *APOE* genotypes (*p* = 0.237, *APOE3*: 70 ± 3.3% coverage vs *APOE4*: 57 ± 10% coverage). These results reflect a slower response to Aβ-42 by *APOE4* microglia, suggesting an altered mechanism associated with initial responses to Aβ seeding. Internalization of Aβ peptide in microglial processes was observed in acute brain slices incubated for 6 h (Fig. 6C).

To test if P2RY12 plays a role in the observed microglial response to Aβ seeding, we incubated acute brain slices in 10 μM PSB0739, a selective P2RY12 inhibitor, for 30 min prior to Aβ injection. We found that microglial process motility towards the peptide deposit was completely disrupted by the inhibition of P2RY12 (*n* = 2 APOE3, *n* = 2 APOE4) (Fig. 6D). Thus, the ATP receptor P2RY12 is necessary for the movement of microglia processes in response to Aβ.

## Discussion

Microglia play a pivotal role in maintaining brain homeostasis under physiological and pathological conditions [10, 34]. Large-scale transcriptomic and functional studies have identified microglia as a potential therapeutic target for neurodegenerative disorders [7, 35–38]. Aging and *APOE4* are the strongest risk factors for AD, but how they affect homeostatic microglia function remains unclear. In this study, we identified alterations in homeostatic and injury-induced microglial behavior associated with *APOE4* in a preclinical mouse model lacking overt AD pathology. We found decreased spontaneous and responsive process motilities in *APOE4* microglia, which are associated with the surveillance of brain parenchyma and the response to damage, respectively. These microglial phenotypes associated with *APOE4* were exacerbated with aging. We also found that processes from *APOE4* microglia surround newly injected Aβ-42 less than *APOE3* microglia do, and the entire response is disrupted upon P2RY12 inhibition. This analysis was done in the cortex, given the early accumulation of Aβ deposits there. By examining microglial function under the physiological condition in the absence of AD pathology, we identified microglial behaviors associated with *APOE4* and aging that could account for the predisposition to AD.

Microglial dynamics play a key role in many physiological functions [5, 6, 39]. Investigating how risk factors such as *APOE4* affect brain functions prior AD pathology may shed light on mechanisms that predispose the brain to neurodegeneration. The role of *APOE* genotype in AD pathology has been extensively explored through large-scale transcriptomic studies in mouse models, human brains, and iPSC-derived microglia [40–44]. Microglia from *APOE4* carriers AD patients had downregulation in pathways associated with Aβ clearance and a proinflammatory transcriptomic profile [40]. Work in iPSCs showed that *APOE4* microglia had a proinflammatory transcription profile and reduced Aβ phagocytosis, suggesting that *APOE4* impairs microglial function [42]. In iPSC-derived microglia, *APOE4* was also associated with dysregulation of lipid metabolism characterized by the accumulation of lipid droplets [43]. *APOE4* microglia isolated from targeted replacement mouse brains are proglycolytic due to increased expression of Hif1α and disrupted tricarboxylic acid cycle [41]. Lipidome analysis of microglia isolated from mice expressing human APOE variants shows aberrant lipid metabolism in *APOE4* microglia [45]. Together, these studies suggest that *APOE4* predisposes to AD pathogenesis through dysregulated immunometabolism. Dysregulation of lipid metabolism and neuroinflammation converge with transcriptional profiles associated with alteration of microglial function, such as chemotaxis and phagocytosis [46, 47]. With the large body of transcriptomic data available, it is imperative to validate the functional impact of *APOE4* in studies that focus on microglial behaviors prior to AD pathology. A recent study in iPSC-derived microglia found that *APOE4* genotype induces the accumulation of lipid droplets and impairs surveillance [48]. In our study, we showed that *APOE4* microglia have functional deficits exhibited by a decrease in brain surveillance and a slower response to chemotactic cues associated with acute damage. Our results complement data from iPSCs [48], as our model resembles more closely normal physiological conditions, including an environment with the basal levels of APOE released by astrocytes. We identified microglial phenotypes that are consistent with a proinflammatory transcriptomic profile.

We characterized microglial dynamics associated with *APOE4* genotype in mice globally expressing human *APOE*. In recent work with human *APOE3* or *APOE4* expression only in microglia [49], dynamic functions were studied using in-vivo two-photon imaging. In line with our findings, microglia expressing *APOE4* had impaired surveillance, and, after focal injury, microglial processes moved slower toward the lesion site compared to *APOE3*-expressing cells. These results suggest that the *APOE4* genotype effects on microglial surveillance and responsiveness to damages are cell autonomous. It requires further investigation to determine whether microglial or astrocytic-derived APOE has a stronger impact on microglial homeostatic behaviors and the extent to which this impact is exacerbated by APOE isoform. Deletion of *APOE4* in astrocytes in a mouse model of tauopathy showed upregulation of homeostatic microglial genes, i.e. P2RY12, downregulation of DAM genes, and decreased synaptic phagocytosis [50]. These results suggest that astrocytic APOE influences the microglial molecular signature and function during AD pathology. Astrocytic and microglial APOE have different biochemical properties: microglial APOE is more glycosylated [51] while astrocytic APOE is more lipidated [52]. In addition, APOE isoforms affect post-translational modification, i.e., APOE4 proteins are less lipidated [53, 54] and glycosylated [55] than APOE3. It is possible that both the difference in biochemical properties of APOE isoforms as well as cell-specific APOE contributes to the alteration of microglial immunometabolism influencing homeostatic microglial dynamics.

Microglia exhibit different cellular phenotypes and dynamic behaviors during physiological and pathological conditions [34]. These functional alterations are linked to different neurological disorders [56, 57]. Several bulk RNAseq and single-cell RNAseq studies have identified markers that constitute a signature of homeostatic microglia [10, 58]. Downregulation of these signature genes, including the ATP receptor P2RY12, is associated with altered microglial homeostasis and is consistent with the progression of AD [7, 59, 60]. We demonstrated that *APOE4* microglia expressed less P2RY12 than *APOE3* microglia, particularly in their processes, results that are consistent with bulk and single-cell RNAseq from *APOE4* mice and iPSC-derived microglia [41, 44, 48]. P2RY12 expression is regarded as a marker of homeostatic or non-activated microglia; however, P2RY12 is necessary for microglial chemotaxis and process motility in response to neuronal damage [11, 12, 61, 62]. Consistent with this notion, we identified chemotactic defects associated with *APOE4* genotype through live tissue imaging. We showed that *APOE4* microglia have a slower response to ATP, a chemotactic cue of acute neuronal damage. These results suggest *APOE4* genotype alters microglia homeostasis, affecting the expression of the P2RY12 receptor, which is necessary for injury-induced dynamic behavior. We proposed that the reduced homeostatic function of *APOE4* microglia results in decreased surveillance of brain parenchyma and inefficient response to acute damages that, over time, lead to larger, chronic damages of neurodegeneration.

During normal aging, microglia undergo drastic changes in transcriptomics, microglial function, decreased phagocytosis, migration, and increased release of inflammatory cytokines associated with reduced homeostatic capacity [63]. In the present study, our control group, *APOE3* microglia, exhibited an age-associated decrease in surveillance of brain parenchyma and slower injury-induced response, consistent with aging studies of cortical and retinal microglia in wild-type animals [64, 65]. Bulk- and single-cell sequencing analysis shows that *APOE4* and aging intractably downregulate transcriptional signatures associated with homeostatic microglia, including P2RY12, and upregulate pathways associated with inflammation [41, 44]. Consistent with this notion, we show that *APOE4-*associated decreased response to ATP is consistent in 12- and 21-month-old mice and that aging further exacerbates this effect. Although we did not detect an effect of *APOE* genotype on spontaneous motility in 12- and 21-month-olds, we showed that in *APOE4* microglia, aging decreases the velocity movement of processes in response to ATP and spontaneous motility. These results suggest that the magnitude of age-associated alteration on microglial behaviors will affect microglia differently depending on their *APOE* genotype. The decreased brain surveillance and response to acute damage in aged microglia might contribute to their loss of neuroprotective function. Microglia expressing *APOE4* may lose their protective function at an earlier age, predisposing the brain to neuronal degeneration.

The entorhinal cortex is a primary site of AD pathogenesis, exhibiting early changes in the accumulation of Aβ plaques and neurofibrillary tangles [31]. Several mechanisms influence microglia surveillance including P2RY13 [59] and neuronal network activity [66], and microglia manifest brain region-dependent heterogeneity in their phenotype [67]. We observed that in 6-month-old *APOE3* brains, EC microglia have a more ramified morphology and display higher surveillance compared to CA1 microglia but have no region-associated differences in response to ATP. However, with aging, the only region-associated difference in microglial dynamics detected was in process velocity, with EC microglia responding to ATP more slowly than CA1 microglia. In *APOE3* brains, the spontaneous motility of EC microglia decreased to levels observed in *APOE4* microglia at 12- and 21 months old, suggesting that EC microglia are more susceptible to the effects of aging on microglial dynamics. These brain region-associated differences in microglia dynamics might be due to local cues coming from astrocytes and neurons affecting microglial gene expression. For example, EC neurons exhibit hyperexcitability due to increased spike firing rate [68–70]. Astrocytes, besides secreting cholesterol and growth factors necessary for microglial viability and microglial function [15, 71], have region-specific roles controlling brain function during health and disease [72, 73]. It is possible that epigenetic regulation of microglial functions makes EC more susceptible to the effects of aging and AD pathology.

In humans, the *APOE4* allele is associated with the presence of amyloid at an early age [74] and increased amyloid burden [75, 76]. It remains unknown how microglia react during the early stages of amyloid seeding. In this study, we showed that *APOE4* microglia extend their processes significantly slower after acute injection of Aβ compared to *APOE3* microglia, and that P2RY12 inhibition prevents the process migration towards Aβ. In a recent *in-vivo* study*,* acute responses of microglia were studied in wild-type animals that received a cortical infusion of Aβ combined with either APOE3 protein (AβE3) or APOE4 protein (AβE4). In the brains that received AβE4 injection, microglia extended their processes to the infusion site significantly slower and had less coverage of the infusion [77]. These results suggest that the presence of modifying APOE isoforms does not need to be expressed from microglia, but perhaps from other cells. It is possible that Aβ-APOE4 interaction and subsequent activation of APOE receptors such as TREM2 [78] are responsible for the deficient response to acute Aβ injection exhibited by *APOE4* microglia. Several studies demonstrate that APOE protein binds to Aβ in an isoform-dependent manner, APOE4 > APOE3 > APOE2 [79, 80]. In addition, TREM2 is involved in microglia migration, phagocytosis, and expression of chemotactic receptors [7, 81, 82]. Recent work in iPSC microglia shows that TREM2 mediates purinergic responses, expression of P2RY12, and process extension [20], suggesting that P2RY12 activity observed here may be influenced by TREM2 activation. Purinergic signaling through P2Y receptors plays a pivotal role in the crosstalk between astrocytes and microglia [83, 84]. Aβ peptide induces Ca^+^ current-mediated release of ATP from astrocytes [85–87]. While several microglial receptors are implicated in Aβ internalization (TREM2, SR-A1, CD33) and downstream activation of inflammatory responses (TLR, complement receptors, Fc receptors) [88], our data show that the ATP receptor P2YR12 is the initial mediator of microglial responsiveness to Aβ. It will be interesting to further explore whether Aβ causes the release of ATP by astrocytes or neurons that guide microglial processes, or the direct activation of TREM2 that causes hypersensitivity of P2RY12.

Long-term imaging in different mouse models of AD for at least 24 weeks shows an increase in Aβ plaque formation at early time points and a significant decline in the rate of plaque formation as Aβ load progresses over time [89–91]. These findings strongly suggest that the initial seeding of Aβ is a critical time point in AD progression. The 5XFAD model of AD amyloid in the presence of *APOE3* (*E3FAD*) or *APOE4* (*E4FAD*) showed that *APOE4* increases plaques number and size, as well as increased microglial reactivity [13, 92, 93]. In a recent examination of microglial *APOE* in vivo [45], human *APOE3* or *APOE4* was conditionally expressed in microglia in APP/PS1 mice. Mice with *APOE4* microglia had more Aβ plaque formation but less microglia surrounding the plaques compared to mice expressing *APOE3* in microglia. Deletion of microglial *APOE4* resulted in reduced Aβ pathology and increased microglial recruitment to plaques [45]. However, these analyses could not address how *APOE* affects the acute responses to Aβ. In the present study, we injected Aβ-42 peptide in brain slices, allowing us to model the initial seeding of Aβ and study the acute microglial response. The slower process migration and decreased coverage of injected Aβ observed in *APOE4* microglia suggest an altered mechanism associated with Aβ uptake compared to APOE3 microglia. In vitro studies have shown that *APOE4* microglia exhibit a less efficient Aβ uptake [42] and have impaired digestion of phagocytized materials [94]. It is imperative to study the interaction between microglia and Aβ peptides over time to investigate how *APOE4*-associated alteration in phagocytosis affects plaque formation in an in vivo system. Thus, understanding how *APOE* genotypes regulate microglial dynamic behaviors as well as their impact on homeostasis and responses to damage may shed light on their role in AD pathogenesis.

### Limitations of the study

Our study has a few limitations. First, acute brain slices affect microglial activation, since they are highly sensitive to tissue disturbances and can adopt an activated phenotype over time [95]. Acute brain slices have been an essential tool for studying dynamic microglial behaviors, as they provide an environment that preserves microglia’s physiological states better than in vitro systems. Thus, the current work is not fully modeling an in vivo environment, and future analyses will be needed to test the in vivo effects of P2RY12 on microglial responses to amyloid across *APOE* genotypes and ages. Second, this work tests the acute effects of microglial responses to damage (including Aβ); more chronic effects would require similar experiments in mouse amyloid models. Third, our mouse model is heterozygous (*CX3CR1*^GFP/+^) for *CX3CR1*; therefore, neuron-microglia interaction mediated by *CX3CL1-CX3CR1* signaling might be somewhat impaired compared to wild-type animals, resulting in decreased cognitive and synaptic function [96]. In addition, recent work [97] suggests that the loss of a single copy of *CX3CR1* leads to alteration in microglial transcriptomics compared to wild-type animals. Although *CX3CR1* is mainly expressed in microglia, expression can also be found in monocytes, subsets of NK, and dendritic cells [98]. Therefore, the potential impact of peripheral deletion *CX3CR1* on brain homeostasis warrants further investigation [99].

## Conclusion

We determined that *APOE4* microglia have decreased surveillance, slower process movement in response to ATP, and decreased coverage of Aβ peptide infusion, processes that are mediated in part by the ATP receptor P2RY12. Future studies with this model would provide more information on the effects of *APOE* genotype on microglia-neuron interaction and its impact on neuronal networks prior to AD pathology. These studies will help to identify the most important microglial behaviors that are altered by *APOE4* and aging and could be used in therapeutic approaches to reduce the risk of AD.

### Supplementary Information

**Additional file 1: Figure S1. **A. Confocal images of GFP (left panel in green) and IBA1 (middle panel in red) in an *APOE3* mouse brain section. Colocalization appears as yellow in the right panel. Scale bar 20 µm. B. Confocal images of GFP (left panel in green) and Tmem119 (middle panel in red) in an *APOE3* mouse brain section. Colocalization appears as yellow in the right panel. Scale bar 20 µm. C. Confocal images of GFP (green), GFAP (red), and APOE (white) in an *APOE3* mouse brain section. Merged image of the three colors is shown in the last panel. Scale bar 20 µm.

**Additional file 2: Figure S2. **A. Quantification of microglia density across brain region *p < 0.05; unpaired two-tailed Student’s t-test. B. Representative images of *APOE3* and *APOE4* microglia in the EC and CA1. Scale bar 20 µm. C. Quantification of endpoints per cell across brain region. unpaired two-tailed Student’s t-test. D. Quantification of total branch length per cell across brain region. *p < 0.05; unpaired two-tailed Student’s t-test. E. Quantification of motility index across brain region. *p < 0.05; unpaired two-tailed Student’s t-test. F. Quantification of process velocity in response to 3 mM ATP across brain region. Unpaired two-tailed Student’s t-test.

**Additional file 3: Figure S3. **A. Representative images of microglia in the entorhinal cortex (EC) and hippocampus (CA1) from 12- and 21-month-old *APOE3* (E3) and *APOE4* (E4) mice. Scale bar 20 µm. B. High magnification images of microglia in the entorhinal cortex (EC) and hippocampus (CA1) from 12- and 21-month-old *APOE3* (E3) and *APOE4* (E4) mice. Scale bar 20 µm. C. Binary overlaps of 12- and 21-months old *APOE3* and *APOE4* microglia in the EC and CA1. Scale bar 10 µm. D. Quantification of process velocity in response to 1 mM ATP across brain region in 12- months old mice. unpaired two-tailed Student’s t test. E. Quantification of process velocity in response to 1 mM ATP across brain region in 21- months old mice. Unpaired two-tailed Student’s t test.

## Data Availability

All data generated in this study are included in this article. Data and materials are available to any interested parties upon request.

## References

[1] 2022 Alzheimer's disease facts and figures. Alzheimers Dement. 2022;18(4), 700–789. 10.1002/alz.12638. 10.1002/alz.12638 35289055

[2] Bu G (2009). Apolipoprotein E and its receptors in Alzheimer's disease: pathways, pathogenesis and therapy. Nat Rev Neurosci.

[3] Flowers SA, Rebeck GW (2020). APOE in the normal brain. Neurobiol Dis.

[4] Liang X, Wu H, Colt M, Guo X, Pluimer B, Zeng J, Dong S, Zhao Z (2021). Microglia and its Genetics in Alzheimer's Disease. Curr Alzheimer Res.

[5] Fernandez CG, Hamby ME, McReynolds ML, Ray WJ (2019). The Role of APOE4 in Disrupting the Homeostatic Functions of Astrocytes and Microglia in Aging and Alzheimer's Disease. Front Aging Neurosci.

[6] Sierra A, Paolicelli RC, Kettenmann H (2019). Cien Anos de Microglia: Milestones in a Century of Microglial Research. Trends Neurosci.

[7] Keren-Shaul, H., Spinrad, A., Weiner, A., Matcovitch-Natan, O., Dvir-Szternfeld, R., Ulland, T. K., David, E., Baruch, K., Lara-Astaiso, D., Toth, B., Itzkovitz, S., Colonna, M., Schwartz, M., & Amit, I. (2017). A Unique Microglia Type Associated with Restricting Development of Alzheimer's Disease. Cell, 169(7), 1276–1290 e1217. 10.1016/j.cell.2017.05.018. 10.1016/j.cell.2017.05.018 28602351

[8] Rangaraju S, Dammer EB, Raza SA, Rathakrishnan P, Xiao H, Gao T, Duong DM, Pennington MW, Lah JJ, Seyfried NT, Levey AI (2018). Identification and therapeutic modulation of a pro-inflammatory subset of disease-associated-microglia in Alzheimer's disease. Mol Neurodegener.

[9] Zhang, B., Gaiteri, C., Bodea, L. G., Wang, Z., McElwee, J., Podtelezhnikov, A. A., Zhang, C., Xie, T., Tran, L., Dobrin, R., Fluder, E., Clurman, B., Melquist, S., Narayanan, M., Suver, C., Shah, H., Mahajan, M., Gillis, T., Mysore, J., . . . Emilsson, V. (2013). Integrated systems approach identifies genetic nodes and networks in late-onset Alzheimer's disease. Cell, 153(3), 707–720. 10.1016/j.cell.2013.03.030. 10.1016/j.cell.2013.03.030 PMC3677161 23622250

[10] Butovsky O, Weiner HL (2018). Microglial signatures and their role in health and disease. Nat Rev Neurosci.

[11] Haynes SE, Hollopeter G, Yang G, Kurpius D, Dailey ME, Gan WB, Julius D (2006). The P2Y12 receptor regulates microglial activation by extracellular nucleotides. Nat Neurosci.

[12] Sipe GO, Lowery RL, Tremblay M, Kelly EA, Lamantia CE, Majewska AK (2016). Microglial P2Y12 is necessary for synaptic plasticity in mouse visual cortex. Nat Commun.

[13] Rodriguez GA, Tai LM, LaDu MJ, Rebeck GW (2014). Human APOE4 increases microglia reactivity at Aβ plaques in a mouse model of Aβ deposition. J Neuroinflammation.

[14] Bennett, F. C., Bennett, M. L., Yaqoob, F., Mulinyawe, S. B., Grant, G. A., Hayden Gephart, M., Plowey, E. D., & Barres, B. A. (2018). A Combination of Ontogeny and CNS Environment Establishes Microglial Identity. Neuron. 98(6), 1170–1183 e1178. 10.1016/j.neuron.2018.05.014. 10.1016/j.neuron.2018.05.014 PMC6023731 29861285

[15] Bohlen, C. J., Bennett, F. C., Tucker, A. F., Collins, H. Y., Mulinyawe, S. B., & Barres, B. A. Diverse Requirements for Microglial Survival, Specification, and Function Revealed by Defined-Medium Cultures. Neuron. 2017;94(4), 759–773 e758. 10.1016/j.neuron.2017.04.043. 10.1016/j.neuron.2017.04.043 PMC5523817 28521131

[16] Butovsky O, Jedrychowski MP, Moore CS, Cialic R, Lanser AJ, Gabriely G, Koeglsperger T, Dake B, Wu PM, Doykan CE, Fanek Z, Liu L, Chen Z, Rothstein JD, Ransohoff RM, Gygi SP, Antel JP, Weiner HL (2014). Identification of a unique TGF-beta-dependent molecular and functional signature in microglia. Nat Neurosci.

[17] Foley KE, Hewes AA, Garceau DT, Kotredes KP, Carter GW, Sasner M, Howell GR (2022). The APOE (epsilon3/epsilon4) Genotype Drives Distinct Gene Signatures in the Cortex of Young Mice. Front Aging Neurosci.

[18] Sepulveda J, Luo N, Nelson M, Ng CAS, Rebeck GW (2022). Independent APOE4 knock-in mouse models display reduced brain APOE protein, altered neuroinflammation, and simplification of dendritic spines. J Neurochem.

[19] Sepulveda-Rodriguez, A., Li, P., Khan, T., Ma, J. D., Carlone, C. A., Bozzelli, P. L., Conant, K. E., Forcelli, P. A., & Vicini, S. (2019). Electroconvulsive Shock Enhances Responsive Motility and Purinergic Currents in Microglia in the Mouse Hippocampus. eNeuro,* 6*(2). 10.1523/ENEURO.0056-19.2019 10.1523/ENEURO.0056-19.2019 PMC6498419 31058213

[20] Jairaman, A., McQuade, A., Granzotto, A., Kang, Y. J., Chadarevian, J. P., Gandhi, S., Parker, I., Smith, I., Cho, H., Sensi, S. L., Othy, S., Blurton-Jones, M., & Cahalan, M. D. (2022). TREM2 regulates purinergic receptor-mediated calcium signaling and motility in human iPSC-derived microglia. Elife, 11. 10.7554/eLife.73021. 10.7554/eLife.73021 PMC8906810 35191835

[21] Avignone E, Milior G, Arnoux I, Audinat E (2019). Electrophysiological Investigation of Microglia. Methods Mol Biol.

[22] Young, K., & Morrison, H. (2018). Quantifying Microglia Morphology from Photomicrographs of Immunohistochemistry Prepared Tissue Using ImageJ. J Vis Exp(136). 10.3791/57648. 10.3791/57648 PMC6103256 29939190

[23] Thevenaz P, Ruttimann UE, Unser M (1998). A pyramid approach to subpixel registration based on intensity. IEEE Trans Image Process.

[24] Schindelin J, Arganda-Carreras I, Frise E, Kaynig V, Longair M, Pietzsch T, Preibisch S, Rueden C, Saalfeld S, Schmid B, Tinevez JY, White DJ, Hartenstein V, Eliceiri K, Tomancak P, Cardona A (2012). Fiji: an open-source platform for biological-image analysis. Nat Methods.

[25] Pinto, B., Morelli, G., Rastogi, M., Savardi, A., Fumagalli, A., Petretto, A., Bartolucci, M., Varea, E., Catelani, T., Contestabile, A., Perlini, L. E., & Cancedda, L. (2020). Rescuing Over-activated Microglia Restores Cognitive Performance in Juvenile Animals of the Dp(16) Mouse Model of Down Syndrome. Neuron, 108(5), 887–904 e812. 10.1016/j.neuron.2020.09.010. 10.1016/j.neuron.2020.09.010 PMC7736620 33027640

[26] Etienne, F., Mastrolia, V., Maroteaux, L., Girault, J. A., Gervasi, N., & Roumier, A. (2019). Two-photon Imaging of Microglial Processes' Attraction Toward ATP or Serotonin in Acute Brain Slices. *J Vis Exp*(143). 10.3791/58788 10.3791/58788 30774130

[27] Zott B, Simon MM, Hong W, Unger F, Chen-Engerer HJ, Frosch MP, Sakmann B, Walsh DM, Konnerth A (2019). A vicious cycle of beta amyloid-dependent neuronal hyperactivation. Science.

[28] Hanisch UK, Kettenmann H (2007). Microglia: active sensor and versatile effector cells in the normal and pathologic brain. Nat Neurosci.

[29] Ransohoff RM, Perry VH (2009). Microglial physiology: unique stimuli, specialized responses. Annu Rev Immunol.

[30] De Biase, L. M., Schuebel, K. E., Fusfeld, Z. H., Jair, K., Hawes, I. A., Cimbro, R., Zhang, H. Y., Liu, Q. R., Shen, H., Xi, Z. X., Goldman, D., & Bonci, A. (2017). Local Cues Establish and Maintain Region-Specific Phenotypes of Basal Ganglia Microglia. Neuron, 95(2), 341–356 e346. 10.1016/j.neuron.2017.06.020. 10.1016/j.neuron.2017.06.020 PMC5754189 28689984

[31] Igarashi KM (2023). Entorhinal cortex dysfunction in Alzheimer's disease. Trends Neurosci.

[32] Davalos D, Grutzendler J, Yang G, Kim JV, Zuo Y, Jung S, Littman DR, Dustin ML, Gan WB (2005). ATP mediates rapid microglial response to local brain injury in vivo. Nat Neurosci.

[33] Chagas, L. D. S., Sandre, P. C., Ribeiro, E. R. N. C. A., Marcondes, H., Oliveira Silva, P., Savino, W., & Serfaty, C. A. (2020). Environmental Signals on Microglial Function during Brain Development, Neuroplasticity, and Disease. Int J Mol Sci, 21(6). 10.3390/ijms21062111. 10.3390/ijms21062111 PMC7139373 32204421

[34] Masuda T, Sankowski R, Staszewski O, Prinz M (2020). Microglia Heterogeneity in the Single-Cell Era. Cell Rep.

[35] Fan YY, Cai QL, Gao ZY, Lin X, Huang Q, Tang W, Liu JH (2017). APOE epsilon4 allele elevates the expressions of inflammatory factors and promotes Alzheimer's disease progression: A comparative study based on Han and She populations in the Wenzhou area. Brain Res Bull.

[36] Krasemann, S., Madore, C., Cialic, R., Baufeld, C., Calcagno, N., El Fatimy, R., Beckers, L., O'Loughlin, E., Xu, Y., Fanek, Z., Greco, D. J., Smith, S. T., Tweet, G., Humulock, Z., Zrzavy, T., Conde-Sanroman, P., Gacias, M., Weng, Z., Chen, H., . . . Butovsky, O. (2017). The TREM2-APOE Pathway Drives the Transcriptional Phenotype of Dysfunctional Microglia in Neurodegenerative Diseases. Immunity, 47(3), 566–581 e569. 10.1016/j.immuni.2017.08.008. 10.1016/j.immuni.2017.08.008 PMC5719893 28930663

[37] Mosher KI, Wyss-Coray T (2014). Microglial dysfunction in brain aging and Alzheimer's disease. Biochem Pharmacol.

[38] Seto M, Weiner RL, Dumitrescu L, Hohman TJ (2021). Protective genes and pathways in Alzheimer's disease: moving towards precision interventions. Mol Neurodegener.

[39] Eyo UB, Bispo A, Liu J, Sabu S, Wu R, DiBona VL, Zheng J, Murugan M, Zhang H, Tang Y, Wu LJ (2018). The GluN2A Subunit Regulates Neuronal NMDA receptor-Induced Microglia-Neuron Physical Interactions. Sci Rep.

[40] Kloske CM, Dugan AJ, Weekman EM, Winder Z, Patel E, Nelson PT, Fardo DW, Wilcock DM (2021). Inflammatory Pathways Are Impaired in Alzheimer Disease and Differentially Associated With Apolipoprotein E Status. J Neuropathol Exp Neurol.

[41] Lee S, Devanney NA, Golden LR, Smith CT, Schwartz JL, Walsh AE, Clarke HA, Goulding DS, Allenger EJ, Morillo-Segovia G, Friday CM, Gorman AA, Hawkinson TR, MacLean SM, Williams HC, Sun RC, Morganti JM, Johnson LA (2023). APOE modulates microglial immunometabolism in response to age, amyloid pathology, and inflammatory challenge. Cell Rep.

[42] Lin, Y. T., Seo, J., Gao, F., Feldman, H. M., Wen, H. L., Penney, J., Cam, H. P., Gjoneska, E., Raja, W. K., Cheng, J., Rueda, R., Kritskiy, O., Abdurrob, F., Peng, Z., Milo, B., Yu, C. J., Elmsaouri, S., Dey, D., Ko, T., . . . Tsai, L. H. (2018). APOE4 Causes Widespread Molecular and Cellular Alterations Associated with Alzheimer's Disease Phenotypes in Human iPSC-Derived Brain Cell Types. Neuron, 98(6), 1294. 10.1016/j.neuron.2018.06.011. 10.1016/j.neuron.2018.06.011 PMC6048952 29953873

[43] Tcw, J., Qian, L., Pipalia, N. H., Chao, M. J., Liang, S. A., Shi, Y., Jain, B. R., Bertelsen, S. E., Kapoor, M., Marcora, E., Sikora, E., Andrews, E. J., Martini, A. C., Karch, C. M., Head, E., Holtzman, D. M., Zhang, B., Wang, M., Maxfield, F. R., . . . Goate, A. M. (2022). Cholesterol and matrisome pathways dysregulated in astrocytes and microglia. *Cell*, *185*(13), 2213–2233 e2225. 10.1016/j.cell.2022.05.017. 10.1016/j.cell.2022.05.017 PMC9340815 35750033

[44] Zhao, N., Ren, Y., Yamazaki, Y., Qiao, W., Li, F., Felton, L. M., Mahmoudiandehkordi, S., Kueider-Paisley, A., Sonoustoun, B., Arnold, M., Shue, F., Zheng, J., Attrebi, O. N., Martens, Y. A., Li, Z., Bastea, L., Meneses, A. D., Chen, K., Thompson, J. W., . . . Bu, G. (2020). Alzheimer's Risk Factors Age, APOE Genotype, and Sex Drive Distinct Molecular Pathways. Neuron, 106(5), 727–742 e726. 10.1016/j.neuron.2020.02.034. 10.1016/j.neuron.2020.02.034 PMC7388065 32199103

[45] Yin, Z., Rosenzweig, N., Kleemann, K. L., Zhang, X., Brandao, W., Margeta, M. A., Schroeder, C., Sivanathan, K. N., Silveira, S., Gauthier, C., Mallah, D., Pitts, K. M., Durao, A., Herron, S., Shorey, H., Cheng, Y., Barry, J. L., Krishnan, R. K., Wakelin, S., . . . Butovsky, O. (2023). APOE4 impairs the microglial response in Alzheimer's disease by inducing TGFbeta-mediated checkpoints. Nat Immunol, 24(11), 1839–1853. 10.1038/s41590-023-01627-6 10.1038/s41590-023-01627-6 PMC10863749 37749326

[46] Chausse B, Kakimoto PA, Kann O (2021). Microglia and lipids: how metabolism controls brain innate immunity. Semin Cell Dev Biol.

[47] Yen JJ, Yu II (2023). The role of ApoE-mediated microglial lipid metabolism in brain aging and disease. Immunometabolism (Cobham).

[48] Victor, M. B., Leary, N., Luna, X., Meharena, H. S., Scannail, A. N., Bozzelli, P. L., Samaan, G., Murdock, M. H., von Maydell, D., Effenberger, A. H., Cerit, O., Wen, H. L., Liu, L., Welch, G., Bonner, M., & Tsai, L. H. (2022). Lipid accumulation induced by APOE4 impairs microglial surveillance of neuronal-network activity. Cell Stem Cell, *29*(8), 1197–1212 e1198. 10.1016/j.stem.2022.07.005. 10.1016/j.stem.2022.07.005 PMC9623845 35931030

[49] Liu, C. C., Wang, N., Chen, Y., Inoue, Y., Shue, F., Ren, Y., Wang, M., Qiao, W., Ikezu, T. C., Li, Z., Zhao, J., Martens, Y., Doss, S. V., Rosenberg, C. L., Jeevaratnam, S., Jia, L., Raulin, A. C., Qi, F., Zhu, Y., . . . Bu, G. (2023). Cell-autonomous effects of APOE4 in restricting microglial response in brain homeostasis and Alzheimer's disease. Nat Immunol, 24(11), 1854–1866. 10.1038/s41590-023-01640-9. 10.1038/s41590-023-01640-9 PMC11980647 37857825

[50] Wang, C., Xiong, M., Gratuze, M., Bao, X., Shi, Y., Andhey, P. S., Manis, M., Schroeder, C., Yin, Z., Madore, C., Butovsky, O., Artyomov, M., Ulrich, J. D., & Holtzman, D. M. (2021). Selective removal of astrocytic APOE4 strongly protects against tau-mediated neurodegeneration and decreases synaptic phagocytosis by microglia. Neuron, 109(10), 1657–1674 e1657. 10.1016/j.neuron.2021.03.024. 10.1016/j.neuron.2021.03.024 PMC8141024 33831349

[51] Lanfranco MF, Sepulveda J, Kopetsky G, Rebeck GW (2021). Expression and secretion of apoE isoforms in astrocytes and microglia during inflammation. Glia.

[52] Huynh TV, Wang C, Tran AC, Tabor GT, Mahan TE, Francis CM, Finn MB, Spellman R, Manis M, Tanzi RE, Ulrich JD, Holtzman DM (2019). Lack of hepatic apoE does not influence early Abeta deposition: observations from a new APOE knock-in model. Mol Neurodegener.

[53] Heinsinger NM, Gachechiladze MA, Rebeck GW (2016). Apolipoprotein E Genotype Affects Size of ApoE Complexes in Cerebrospinal Fluid. J Neuropathol Exp Neurol.

[54] Hu J, Liu CC, Chen XF, Zhang YW, Xu H, Bu G (2015). Opposing effects of viral mediated brain expression of apolipoprotein E2 (apoE2) and apoE4 on apoE lipidation and Aβ metabolism in apoE4-targeted replacement mice. Mol Neurodegener.

[55] Hu Y, Meuret C, Go S, Yassine HN, Nedelkov D (2020). Simple and Fast Assay for Apolipoprotein E Phenotyping and Glycotyping: Discovering Isoform-Specific Glycosylation in Plasma and Cerebrospinal Fluid. J Alzheimers Dis.

[56] Song WM, Colonna M (2018). The Microglial Response to Neurodegenerative Disease. Adv Immunol.

[57] Thion MS, Ginhoux F, Garel S (2018). Microglia and early brain development: An intimate journey. Science.

[58] Deczkowska A, Keren-Shaul H, Weiner A, Colonna M, Schwartz M, Amit I (2018). Disease-Associated Microglia: A Universal Immune Sensor of Neurodegeneration. Cell.

[59] Kyrargyri V, Madry C, Rifat A, Arancibia-Carcamo IL, Jones SP, Chan VTT, Xu Y, Robaye B, Attwell D (2020). P2Y13 receptors regulate microglial morphology, surveillance, and resting levels of interleukin 1beta release. Glia.

[60] Walker, D. G., Tang, T. M., Mendsaikhan, A., Tooyama, I., Serrano, G. E., Sue, L. I., Beach, T. G., & Lue, L. F. (2020). Patterns of Expression of Purinergic Receptor P2RY12, a Putative Marker for Non-Activated Microglia, in Aged and Alzheimer's Disease Brains. Int J Mol Sci, *21*(2). 10.3390/ijms21020678. 10.3390/ijms21020678 PMC7014248 31968618

[61] Csaszar, E., Lenart, N., Cserep, C., Kornyei, Z., Fekete, R., Posfai, B., Balazsfi, D., Hangya, B., Schwarcz, A. D., Szabadits, E., Szollosi, D., Szigeti, K., Mathe, D., West, B. L., Sviatko, K., Bras, A. R., Mariani, J. C., Kliewer, A., Lenkei, Z., . . . Denes, A. (2022). Microglia modulate blood flow, neurovascular coupling, and hypoperfusion via purinergic actions. J Exp Med, 219(3). 10.1084/jem.20211071. 10.1084/jem.20211071 PMC8932534 35201268

[62] Cserep C, Schwarcz AD, Posfai B, Laszlo ZI, Kellermayer A, Kornyei Z, Kisfali M, Nyerges M, Lele Z, Katona I, Adam D (2022). Microglial control of neuronal development via somatic purinergic junctions. Cell Rep.

[63] Yoo HJ, Kwon MS (2021). Aged Microglia in Neurodegenerative Diseases: Microglia Lifespan and Culture Methods. Front Aging Neurosci.

[64] Damani MR, Zhao L, Fontainhas AM, Amaral J, Fariss RN, Wong WT (2011). Age-related alterations in the dynamic behavior of microglia. Aging Cell.

[65] Hefendehl JK, Neher JJ, Suhs RB, Kohsaka S, Skodras A, Jucker M (2014). Homeostatic and injury-induced microglia behavior in the aging brain. Aging Cell.

[66] Liu YU, Ying Y, Li Y, Eyo UB, Chen T, Zheng J, Umpierre AD, Zhu J, Bosco DB, Dong H, Wu LJ (2019). Neuronal network activity controls microglial process surveillance in awake mice via norepinephrine signaling. Nat Neurosci.

[67] Tan YL, Yuan Y, Tian L (2020). Microglial regional heterogeneity and its role in the brain. Mol Psychiatry.

[68] Busche MA, Eichhoff G, Adelsberger H, Abramowski D, Wiederhold KH, Haass C, Staufenbiel M, Konnerth A, Garaschuk O (2008). Clusters of hyperactive neurons near amyloid plaques in a mouse model of Alzheimer's disease. Science.

[69] Jun, H., Bramian, A., Soma, S., Saito, T., Saido, T. C., & Igarashi, K. M. (2020). Disrupted Place Cell Remapping and Impaired Grid Cells in a Knockin Model of Alzheimer's Disease. Neuron, 107(6), 1095–1112 e1096. 10.1016/j.neuron.2020.06.023. 10.1016/j.neuron.2020.06.023 PMC7529950 32697942

[70] Nakazono T, Lam TN, Patel AY, Kitazawa M, Saito T, Saido TC, Igarashi KM (2017). Impaired In Vivo Gamma Oscillations in the Medial Entorhinal Cortex of Knock-in Alzheimer Model. Front Syst Neurosci.

[71] Garland EF, Hartnell IJ, Boche D (2022). Microglia and Astrocyte Function and Communication: What Do We Know in Humans?. Front Neurosci.

[72] Batiuk MY, Martirosyan A, Wahis J, de Vin F, Marneffe C, Kusserow C, Koeppen J, Viana JF, Oliveira JF, Voet T, Ponting CP, Belgard TG, Holt MG (2020). Identification of region-specific astrocyte subtypes at single cell resolution. Nat Commun.

[73] Xin W, Bonci A (2018). Functional Astrocyte Heterogeneity and Implications for Their Role in Shaping Neurotransmission. Front Cell Neurosci.

[74] Jansen, W. J., Ossenkoppele, R., Knol, D. L., Tijms, B. M., Scheltens, P., Verhey, F. R., Visser, P. J., Aalten, P., Aarsland, D., Alcolea, D., Alexander, M., Almdahl, I. S., Arnold, S. E., Baldeiras, I., Barthel, H., van Berckel, B. N., Bibeau, K., Blennow, K., Brooks, D. J., . . . Zetterberg, H. (2015). Prevalence of cerebral amyloid pathology in persons without dementia: a meta-analysis. Jama, 313(19), 1924–1938. 10.1001/jama.2015.4668. 10.1001/jama.2015.4668 PMC4486209 25988462

[75] Rebeck GW, Reiter JS, Strickland DK, Hyman BT (1993). Apolipoprotein E in sporadic Alzheimer's disease: allelic variation and receptor interactions. Neuron.

[76] Schmechel DE, Saunders AM, Strittmatter WJ, Crain BJ, Hulette CM, Joo SH, Pericak-Vance MA, Goldgaber D, Roses AD (1993). Increased amyloid beta-peptide deposition in cerebral cortex as a consequence of apolipoprotein E genotype in late-onset Alzheimer disease. Proc Natl Acad Sci U S A.

[77] Fitz NF, Nam KN, Wolfe CM, Letronne F, Playso BE, Iordanova BE, Kozai TDY, Biedrzycki RJ, Kagan VE, Tyurina YY, Han X, Lefterov I, Koldamova R (2021). Phospholipids of APOE lipoproteins activate microglia in an isoform-specific manner in preclinical models of Alzheimer's disease. Nat Commun.

[78] Atagi Y, Liu CC, Painter MM, Chen XF, Verbeeck C, Zheng H, Li X, Rademakers R, Kang SS, Xu H, Younkin S, Das P, Fryer JD, Bu G (2015). Apolipoprotein E Is a Ligand for Triggering Receptor Expressed on Myeloid Cells 2 (TREM2). J Biol Chem.

[79] Kanekiyo T, Xu H, Bu G (2014). ApoE and Abeta in Alzheimer's disease: accidental encounters or partners?. Neuron.

[80] Wisniewski T, Drummond E (2020). APOE-amyloid interaction: Therapeutic targets. Neurobiol Dis.

[81] Kim SM, Mun BR, Lee SJ, Joh Y, Lee HY, Ji KY, Choi HR, Lee EH, Kim EM, Jang JH, Song HW, Mook-Jung I, Choi WS, Kang HS (2017). TREM2 promotes Abeta phagocytosis by upregulating C/EBPalpha-dependent CD36 expression in microglia. Sci Rep.

[82] Mazaheri, F., Snaidero, N., Kleinberger, G., Madore, C., Daria, A., Werner, G., Krasemann, S., Capell, A., Trumbach, D., Wurst, W., Brunner, B., Bultmann, S., Tahirovic, S., Kerschensteiner, M., Misgeld, T., Butovsky, O., & Haass, C. (2017). TREM2 deficiency impairs chemotaxis and microglial responses to neuronal injury. *Embo Reports*, *18*(7), 1186–1198. 10.15252/embr.201743922. 10.15252/embr.201743922 PMC5494532 28483841

[83] Matejuk A, Ransohoff RM (2020). Crosstalk Between Astrocytes and Microglia: An Overview. Front Immunol.

[84] Xiong Y, Sun S, Teng S, Jin M, Zhou Z (2018). Ca(2+)-Dependent and Ca(2+)-Independent ATP Release in Astrocytes. Front Mol Neurosci.

[85] Jung ES, An K, Hong HS, Kim JH, Mook-Jung I (2012). Astrocyte-originated ATP protects Abeta(1–42)-induced impairment of synaptic plasticity. J Neurosci.

[86] Madeira D, Dias L, Santos P, Cunha RA, Canas PM, Agostinho P (2021). Association Between Adenosine A(2A) Receptors and Connexin 43 Regulates Hemichannels Activity and ATP Release in Astrocytes Exposed to Amyloid-beta Peptides. Mol Neurobiol.

[87] Pham C, Herault K, Oheim M, Maldera S, Vialou V, Cauli B, Li D (2021). Astrocytes respond to a neurotoxic Abeta fragment with state-dependent Ca(2+) alteration and multiphasic transmitter release. Acta Neuropathol Commun.

[88] Doens D, Fernandez PL (2014). Microglia receptors and their implications in the response to amyloid beta for Alzheimer's disease pathogenesis. J Neuroinflammation.

[89] Burgold S, Bittner T, Dorostkar MM, Kieser D, Fuhrmann M, Mitteregger G, Kretzschmar H, Schmidt B, Herms J (2011). In vivo multiphoton imaging reveals gradual growth of newborn amyloid plaques over weeks. Acta Neuropathol.

[90] Burgold S, Filser S, Dorostkar MM, Schmidt B, Herms J (2014). In vivo imaging reveals sigmoidal growth kinetic of beta-amyloid plaques. Acta Neuropathol Commun.

[91] Hefendehl JK, Wegenast-Braun BM, Liebig C, Eicke D, Milford D, Calhoun ME, Kohsaka S, Eichner M, Jucker M (2011). Long-term in vivo imaging of beta-amyloid plaque appearance and growth in a mouse model of cerebral beta-amyloidosis. J Neurosci.

[92] Yang Y, Cudaback E, Jorstad NL, Hemingway JF, Hagan CE, Melief EJ, Li X, Yoo T, Khademi SB, Montine KS, Montine TJ, Keene CD (2013). APOE3, but not APOE4, bone marrow transplantation mitigates behavioral and pathological changes in a mouse model of Alzheimer disease. Am J Pathol.

[93] Youmans KL, Tai LM, Nwabuisi-Heath E, Jungbauer L, Kanekiyo T, Gan M, Kim J, Eimer WA, Estus S, Rebeck GW, Weeber EJ, Bu G, Yu C, Ladu MJ (2012). APOE4-specific changes in Abeta accumulation in a new transgenic mouse model of Alzheimer disease. J Biol Chem.

[94] Machlovi SI, Neuner SM, Hemmer BM, Khan R, Liu Y, Huang M, Zhu JD, Castellano JM, Cai D, Marcora E, Goate AM (2022). APOE4 confers transcriptomic and functional alterations to primary mouse microglia. Neurobiol Dis.

[95] Berki P, Cserép C, Pósfai B, Szabadits E, Környei Z, Kellermayer A, Nyerges M, Wei X, Mody I, Araki K, Beck H. Microglia undergo rapid phenotypic transformation in acute brain slices but remain essential for neuronal synchrony. bioRxiv. 2022:2022–04. 10.1101/2022.04.12.487998.

[96] Rogers JT, Morganti JM, Bachstetter AD, Hudson CE, Peters MM, Grimmig BA, Weeber EJ, Bickford PC, Gemma C (2011). CX3CR1 deficiency leads to impairment of hippocampal cognitive function and synaptic plasticity. J Neurosci.

[97] Gyoneva, S., Hosur, R., Gosselin, D., Zhang, B., Ouyang, Z., Cotleur, A. C., Peterson, M., Allaire, N., Challa, R., Cullen, P., Roberts, C., Miao, K., Reynolds, T. L., Glass, C. K., Burkly, L., & Ransohoff, R. M. (2019). Cx3cr1-deficient microglia exhibit a premature aging transcriptome. *Life Sci Alliance*,* 2*(6). 10.26508/lsa.201900453. 10.26508/lsa.201900453 PMC6892408 31792059

[98] Jung S, Aliberti J, Graemmel P, Sunshine MJ, Kreutzberg GW, Sher A, Littman DR (2000). Analysis of fractalkine receptor CX(3)CR1 function by targeted deletion and green fluorescent protein reporter gene insertion. Mol Cell Biol.

[99] Garre JM, Silva HM, Lafaille JJ, Yang G (2017). CX3CR1(+) monocytes modulate learning and learning-dependent dendritic spine remodeling via TNF-alpha. Nat Med.

