# Supplementary figures and images for: APOE4 genotype and aging impair injury-induced microglial behavior in brain slices, including toward Aβ, through P2RY12
Source: Mol Neurodegener. 2024 Mar 11;19:24. doi: 10.1186/s13024-024-00714-y (PMC10929239; doi:10.1186/s13024-024-00714-y)

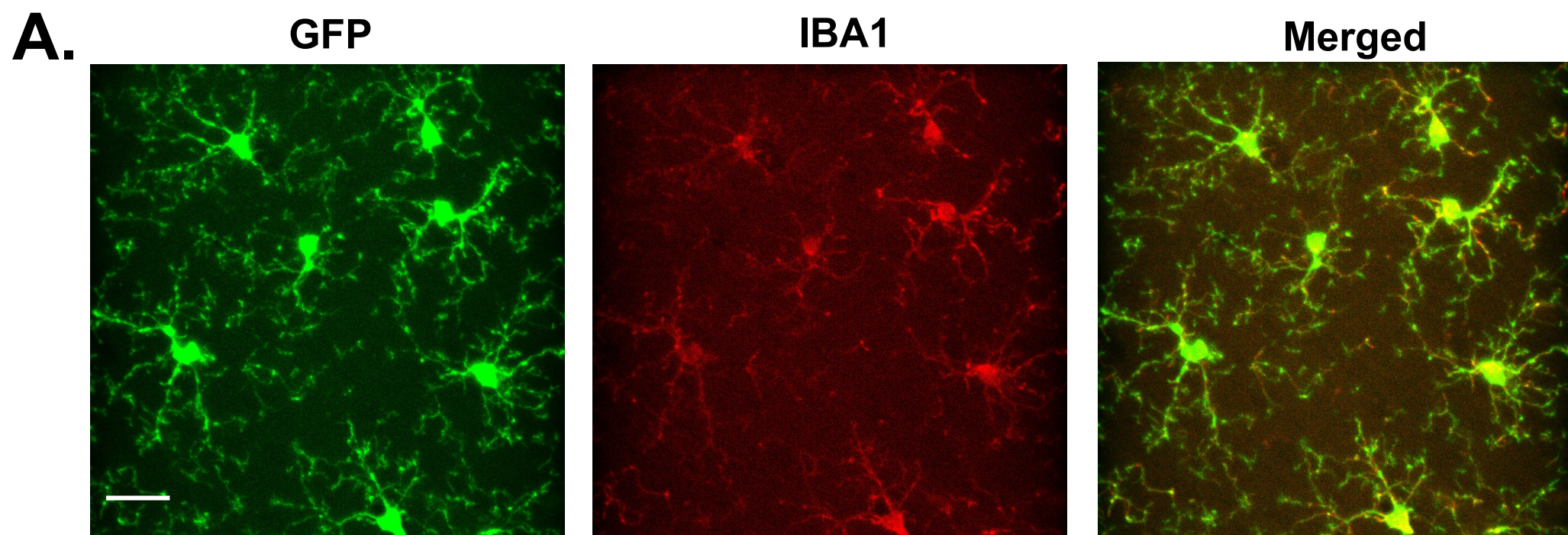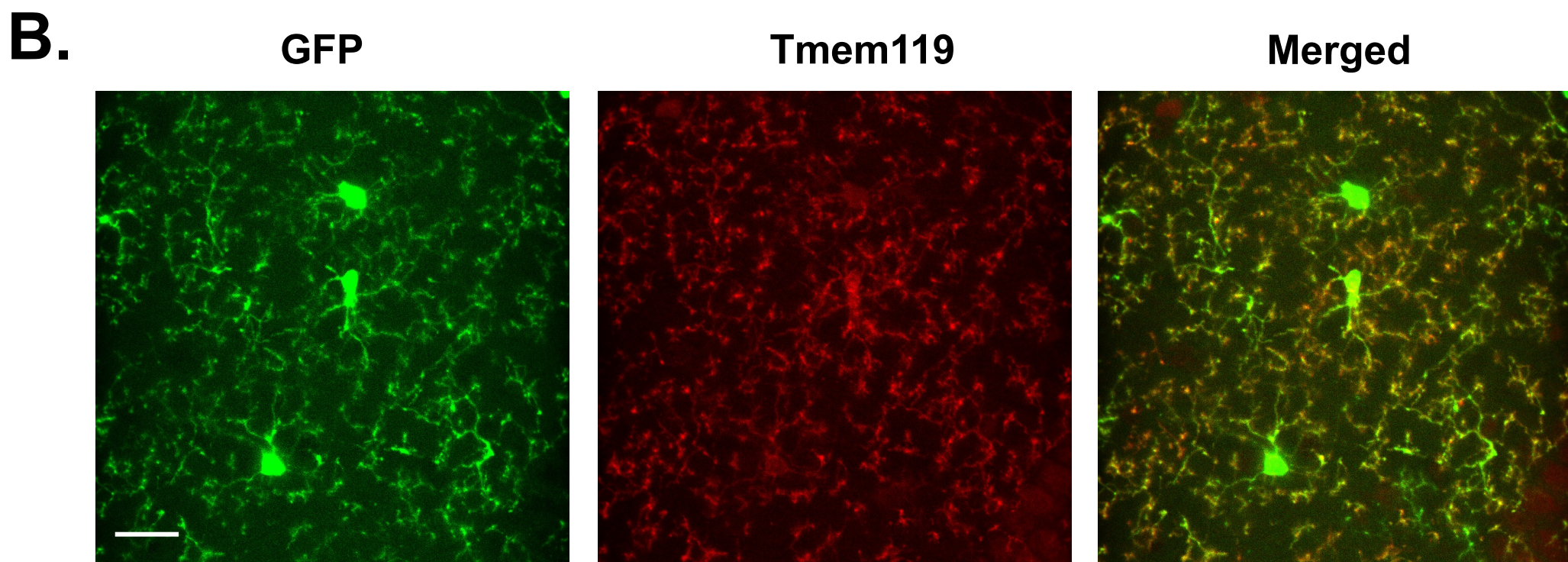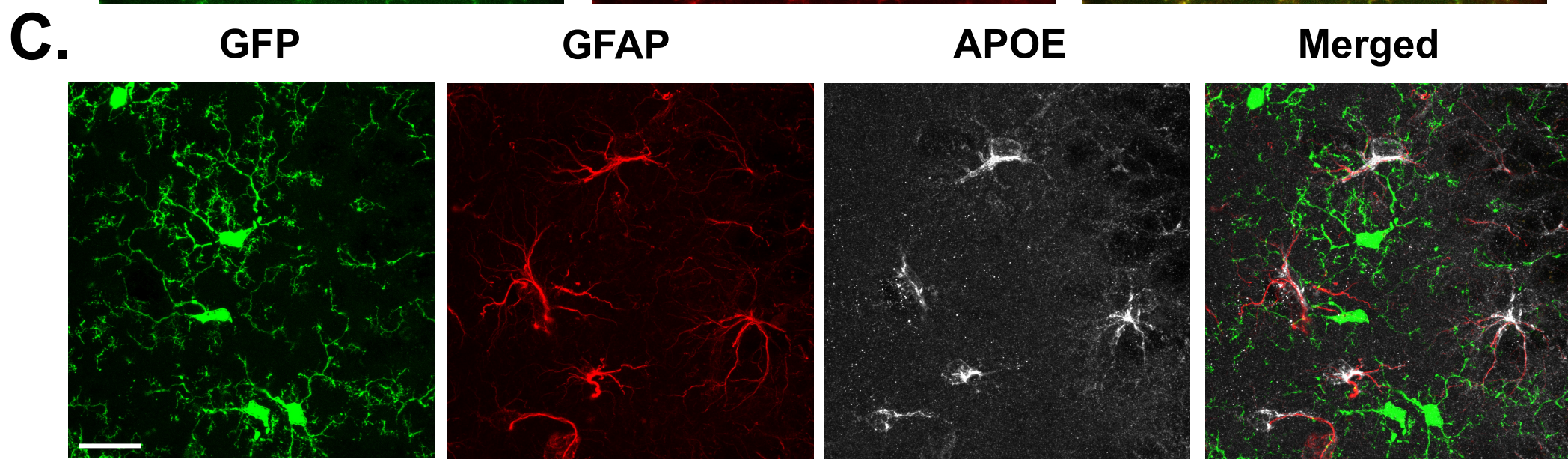

Supplement: Supplementary file 1 — Additional file 1: Figure S1. A. Confocal images of GFP (left panel in green) and IBA1 (middle panel in red) in an APOE3 mouse brain section. Colocalization appears as yellow in the right panel. Scale bar 20 µm. B. Confocal images of GFP (left panel in green) and Tmem119 (middle panel in red) in an APOE3 mouse brain section. Colocalization appears as yellow in the right panel. Scale bar 20 µm. C. Confocal images of GFP (green), GFAP (red), and APOE (white) in an APOE3 mouse brain section. Merged image of the three colors is shown in the last panel. Scale bar 20 µm. [file 13024_2024_714_MOESM1_ESM.pdf]

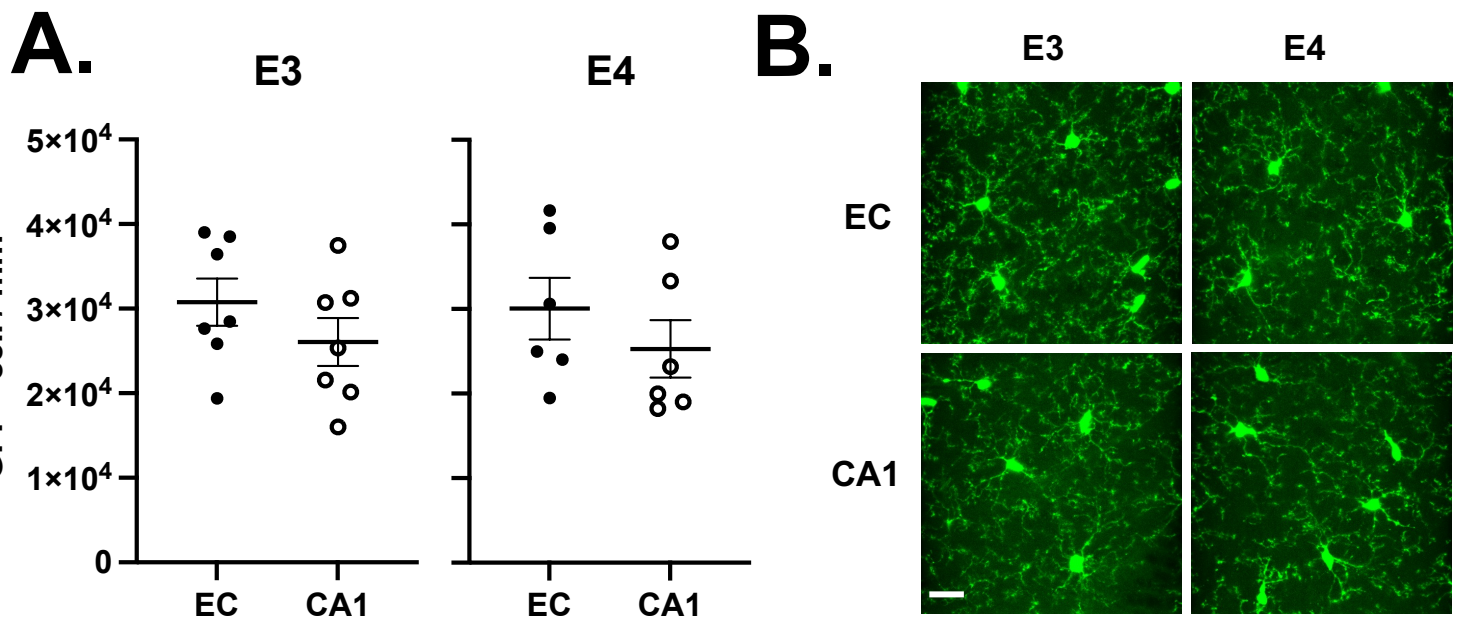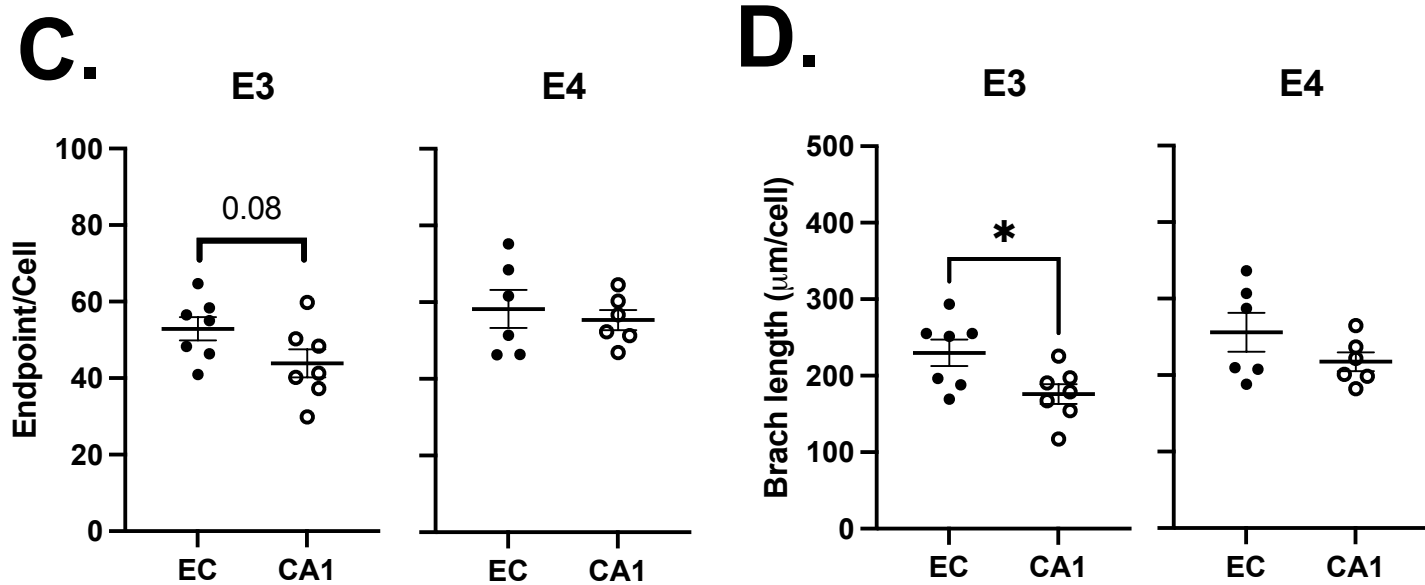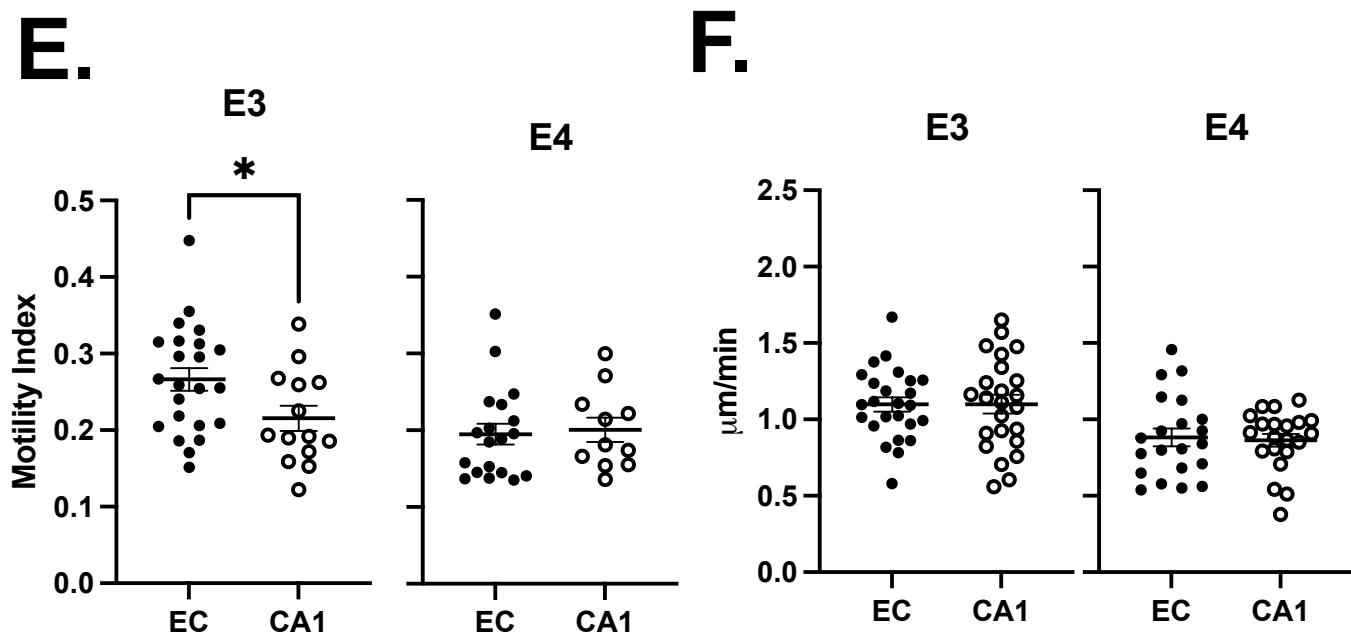

Supplement: Supplementary file 2 — Additional file 2: Figure S2. A. Quantification of microglia density across brain region *p < 0.05; unpaired two-tailed Student’s t-test. B. Representative images of APOE3 and APOE4 microglia in the EC and CA1. Scale bar 20 µm. C. Quantification of endpoints per cell across brain region. unpaired two-tailed Student’s t-test. D. Quantification of total branch length per cell across brain region. *p < 0.05; unpaired two-tailed Student’s t-test. E. Quantification of motility index across brain region. *p < 0.05; unpaired two-tailed Student’s t-test. F. Quantification of process velocity in response to 3 mM ATP across brain region. Unpaired two-tailed Student’s t-test. [file 13024_2024_714_MOESM2_ESM.pdf]

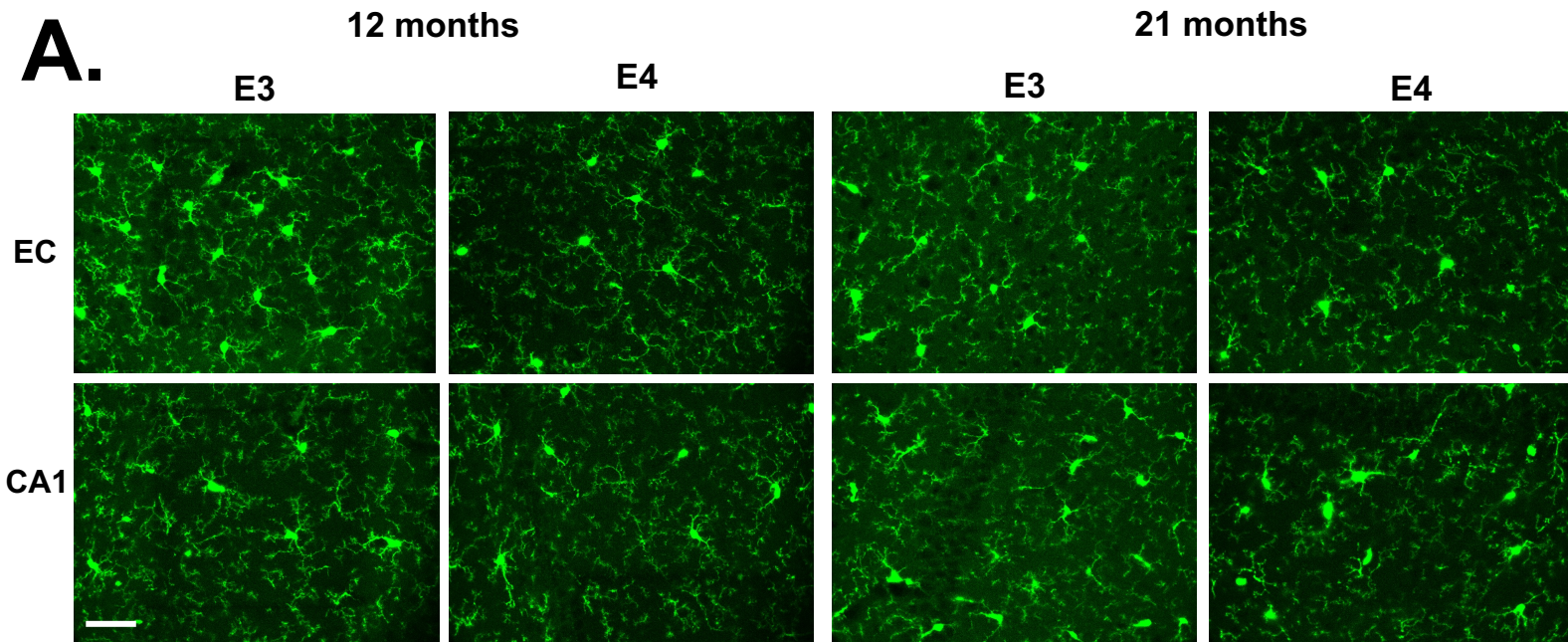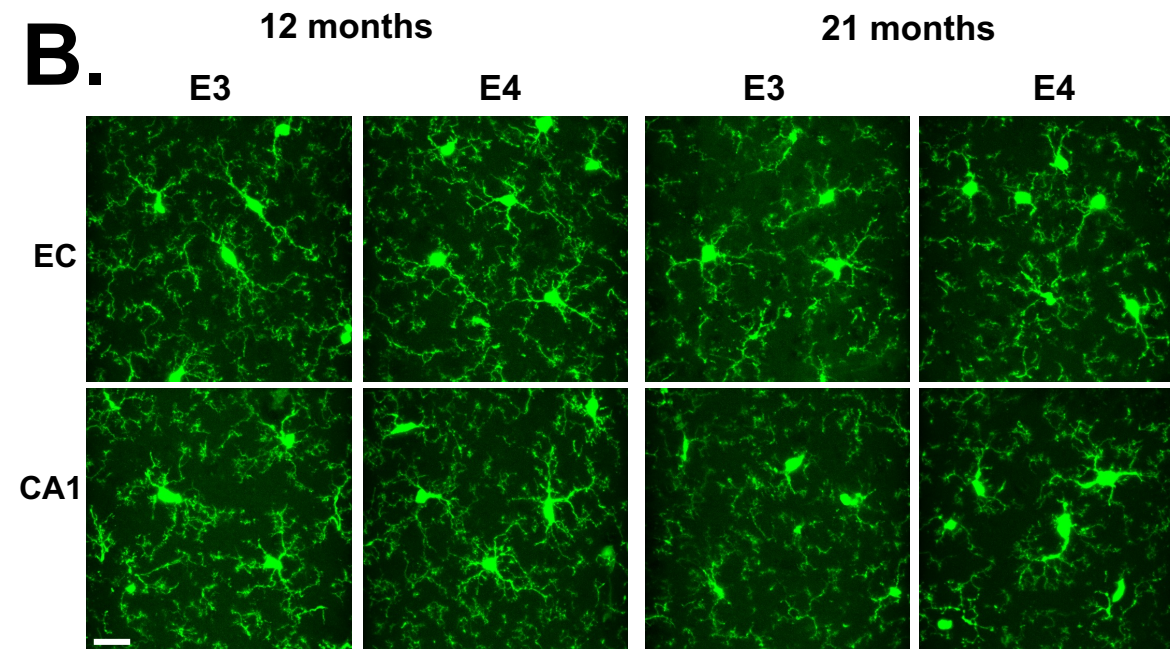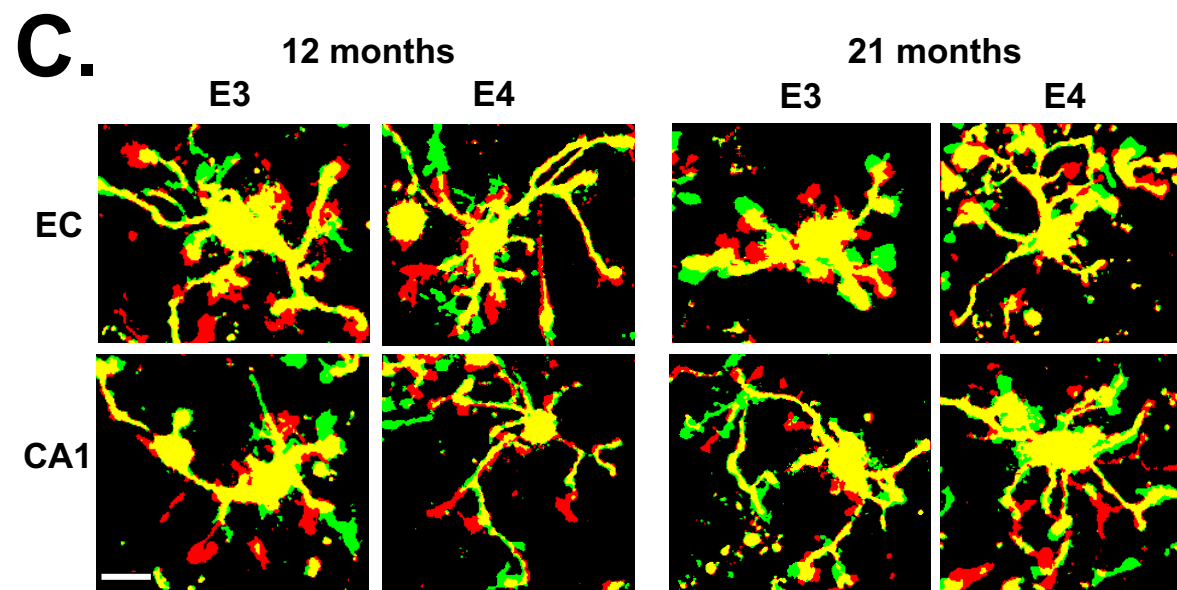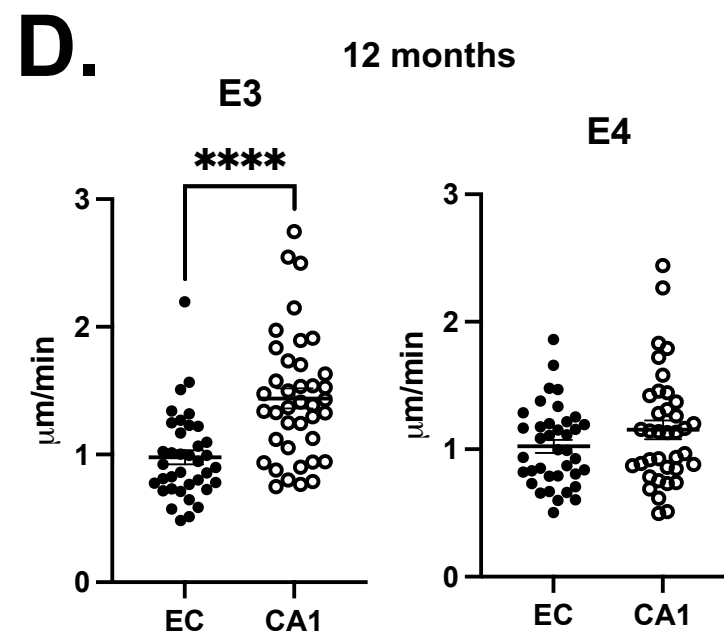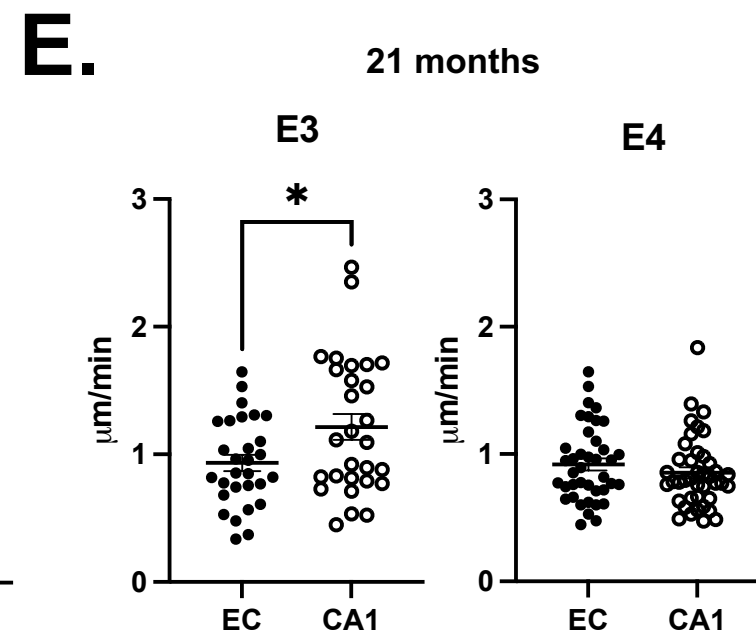

Supplement: Supplementary file 3 — Additional file 3: Figure S3. A. Representative images of microglia in the entorhinal cortex (EC) and hippocampus (CA1) from 12- and 21-month-old APOE3 (E3) and APOE4 (E4) mice. Scale bar 20 µm. B. High magnification images of microglia in the entorhinal cortex (EC) and hippocampus (CA1) from 12- and 21-month-old APOE3 (E3) and APOE4 (E4) mice. Scale bar 20 µm. C. Binary overlaps of 12- and 21-months old APOE3 and APOE4 microglia in the EC and CA1. Scale bar 10 µm. D. Quantification of process velocity in response to 1 mM ATP across brain region in 12- months old mice. unpaired two-tailed Student’s t test. E. Quantification of process velocity in response to 1 mM ATP across brain region in 21- months old mice. Unpaired two-tailed Student’s t test. [file 13024_2024_714_MOESM3_ESM.pdf]
